# Main Bioactive Components and Their Biological Activities from Natural and Processed Rhizomes of *Polygonum sibiricum*

**DOI:** 10.3390/antiox11071383

**Published:** 2022-07-17

**Authors:** Shih-Chi Chen, Chang-Syun Yang, Jih-Jung Chen

**Affiliations:** 1 Department of Pharmacy, School of Pharmaceutical Sciences, National Yang Ming Chiao Tung University, Taipei 112304, Taiwan; asd19981214.ps10@nycu.edu.tw (S.-C.C.); tim0619@nycu.edu.tw (C.-S.Y.); 2 Department of Medical Research, China Medical University Hospital, China Medical University, Taichung 404332, Taiwan

**Keywords:** *Polygonatum sibiricum*, coumarin, flavone glycosides, bioactive components, antioxidant effect, anti-α-glucosidase effect, anti-acetylcholinesterase effect, anti-inflammatory activity, molecular docking

## Abstract

*Polygonatum sibiricum* (Asparagaceae) is often used as an herbal drug in the traditional medicine of Southeast Asia. Its rhizome, called “Huang Jing”, is used in traditional Chinese medicine as an immune system stimulant, hypolipidemic agent, anti-aging agent, anti-fatigue agent, and cardiovascular protectant. We investigated the antioxidant, anti-acetylcholinesterase (AChE), anti-inflammatory, and anti-α-glucosidase effects of various solvent extracts and major bioactive components of *Polygonatum sibiricum* (PS) and processed *Polygonatum sibiricum* (PPS). Dichloromethane extract of PS showed stronger antioxidant effects by DPPH, ABTS, and FRAP assays, and EtOAc extract displayed relatively high antioxidant activity by a superoxide radical scavenging test. Moreover, acetone, EtOAc, and dichloromethane extracts displayed a significant anti-α-glucosidase effect. EtOH and CH_2_Cl_2_ extracts showed effective AChE inhibitory activity. In addition, dichloromethane extract showed the best inhibition against lipopolysaccharide (LPS)-induced nitric oxide (NO) accumulation in RAW264.7 macrophages. HPLC analysis was used to investigate and compare the content of major active components of various solvent extracts of PS and PPS. Rutin showed the most effective scavenging of DPPH and ABTS free radicals, while scopoletin and isoquercetin displayed the strongest anti-α-glucosidase and anti-AChE effect, respectively. Rutin showed the best inhibition against LPS-induced NO production and also inhibited inducible nitric oxide synthase (iNOS) expression in Western blot. The molecular docking of AChE and iNOS revealed that active components could have a better antagonistic effect than positive controls (common inhibitors). This study shows that the active extracts and components of *Polygonatum sibiricum* have the potential to be further developed as a natural anti-AChE, anti-α-glucosidase, antioxidant and anti-inflammatory agent.

## 1. Introduction

The dried rhizome of *Polygonum* *sibiricum* is known as “Huang Jing” in Taiwan and is listed in the Chinese Pharmacopoeia (2015 edition). According to the application, the raw medicinal herbs normally must be processed before use in clinical prescriptions. *P. sibiricum* can be classified as fresh PS, dried PS (PS) and processed PS (PPS) according to different processing methods. It is well known that nine cycles of steaming and sun-drying are a traditional processing method for processed PS [1]. Diverse flavones [2], homoisoflavanones [3], alkaloids [4], steroid saponins [5], triterpenoid saponins [6], polysaccharides [7] and their derivatives were isolated from this species in past studies. Past studies also showed that this herb exhibited anti-aging [7,8], anti-inflammatory [9,10], anti-osteoporotic [11,12], immune enhancing [13,14], neuroprotective [15], anti-diabetic [16], anticancer [17], and sleep-promoting [18] activities.

5-Hydroxymethylfurfural (5-HMF) is the most important component that changes before and after processing *Polygonatum sibiricum* and is one of the representative products of the caramelization reaction and Maillard reaction during PS processing. Long-term steaming converts reducing sugar into 5-HMF, which can be used as the main basis to distinguish PS from PPS, and the number of 5-HMFs of processed PS are several times higher than those of PS [19]. The number of 5-HMF inspectable in materials is indirectly related to the heat load used during the processing of carbohydrate-rich products [20].

Carbohydrates are an important part of our daily diet. Most carbohydrates are digested by salivary and pancreatic amylases and further broken down into monosaccharides by enzymes (mainly for α-glucosidase) in the brush border membrane of enterocytes [21]. α-Glucosidase inhibitors have been considered to be the most effective in reducing postprandial hyperglycemia of all antidiabetic drugs used to treat type 2 diabetes [22].

Oxidative stress plays a crucial role in Alzheimer’s disease (AD) and other related neurological diseases, as well as diabetes. Intracellular oxidative stress is caused by the production of reactive oxygen species and reactive nitrogen species and the poor antioxidant capacity of cells. Therefore, excess reactive oxygen species and reactive nitrogen species can lead to protein and nucleic acid damage, which has been shown to be directly related to diabetes and AD. Oxidative stress increases the production of advanced glycation end products through sugar oxidation and lipid peroxidation. Advanced glycation end products and lipid peroxidation products are abundant in diabetes and AD and serve as markers for both diseases [23]. Endogenously or exogenously introduced antioxidants and inhibitors of advanced glycation end products can counteract the deleterious effects of reactive oxygen species/nitrogen species, thereby preventing or treating the onset of these devastating lesions. Since the primary enzyme in AD pathogenesis is acetylcholinesterase, inhibition of AChE enhances choline signaling in this pathway and reduces the symptoms of AD. The use of natural substances such as chlorogenic acid can lessen the risk of AD. Chlorogenic acid is the main polyphenol component in hawthorn, which has the effect of protecting the heart, inhibiting lipid peroxidation, scavenging free radicals, anti-tumor, and anti-AChE [24].

Nitric oxide (NO) is a free radical involved in the regulation of many physiological processes, such as vascular relaxation, neurotransmission, platelet aggregation, and immune regulation. NO is produced by nitric oxide synthase (NOS) from molecular oxygen and l-arginine, yielding l-citrulline as a by-product. When cytotoxins produced by inflammatory substances are present in the environment, the iNOS gene is stimulated, and its expression is elevated, thereby enabling iNOS to promote the production of nitric oxide [25].

Flavonoids are considered health-promoting substances, some of which have anti-inflammatory and antioxidant properties. Flavonoids are abundantly present in vegetables, fruits, red wine and tea, and the flavonoid mostly consumed is quercetin. Quercetin has a variety of biological effects, such as hypoglycemic and anti-inflammatory activities [26,27].

**Figure 1:**
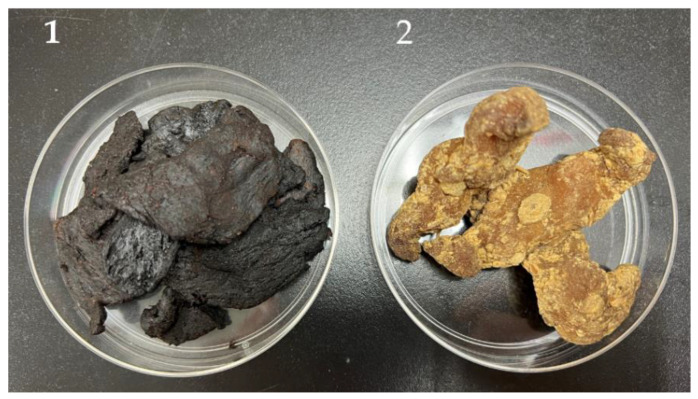
Processed (1) and natural (2) rhizomes of *Polygonatum sibiricum* were used in this study.

A comparative study of the biological activities of different solvent extracts and their components of natural and processed rhizomes of *P. sibiricum* (Figure 1) has not been carried out in the past. Therefore, this study will explore the relationship between “various solvent extracts and their active components” and “antioxidant, anti-AChE, anti-α-glucosidase and anti-inflammatory effects”. The molecular docking research of bioactive compounds and related enzyme proteins is also discussed here.

## 2. Materials and Methods

### 2.1. Chemicals and Reagents

Folin–Ciocalteau’s reagent, bovine serum albumin, ABTS, acarbose, sodium dodecyl sulfate, EDTA, sodium bicarbonate, α-glucosidase, iNOS, TNF-α, IL-6, acetylcholinesterase, lipopolysaccharide, acetylcholine iodide, DTNB, TPTZ, chlorogenic acid, DMSO, and Trolox were purchased from Sigma-Aldrich (St. Louis, MO, USA). Glycine was purchased from J.T.Baker (Phillipsburg, NJ, USA). DPPH, phenazine methosulphate, and nitroblue tetrazolium were supplied from Tokyo Chemical Industry Co., Ltd. (Tokyo, Japan). Thiazolyl blue tetrazolium bromide was purchased from MedChemExpress (Monmouth Junction, NJ, USA). Butyl hydroxytoluene, nicotinamide adenine dinucleotide, sodium acetate, and potassium acetate were supplied from Acros Organics (Geel, Belgium). Ammonium persulfate and TEMED were purchased from Bio-Rad (Hercules, CA, USA). Tween20 was purchased from Merck (Darmstadt, Germany). Acetic acid was supplied from Macron Fine Chemicals (Center Valley, PA, USA). Iron (III) chloride, aluminum chloride, as well as *p*-Nitro-phenyl-α-D-glucopyranoside were purchased from Alfa Aesar (Lancashire, UK). Disodium hydrogenphosphate, sodium carbonate, potassium peroxodisulfate, and potassium dihydrogenphosphate were obtained from SHOWA Chemical Co. (Sun Valley, CA, USA).

### 2.2. Preparation of P. sibiricum Extracts

The rhizomes of *P. sibiricum* were purchased from Nantou, Taiwan, in January 2022, and the processed rhizomes of *P. sibiricum* were supplied from Changsheng Chinese medicine shop, Taipei city, Taiwan, in August 2021 and identified by Prof. J.-J. Chen. The voucher specimens were stored in the Department of Pharmacy, National Yang Ming Chiao Tung University, Taipei, Taiwan. First, the rhizomes of *P. sibiricum* (15 g) were soaked in 150 mL of different solvents (water, MeOH, EtOH, acetone, EtOAc, and dichloromethane) and shaken on an orbital shaker for 24 h at room temperature. Subsequently, the extracts were filtered through filter paper (Whatman No. 1) and concentrated using a rotary evaporator at 38 °C. Finally, all extracts were stored at −20 °C until further experiments.

### 2.3. Preparation of Bioactive Components

The dried rhizomes (1.0 kg) of *Polygonatum sibiricum* were extracted 3 times for 3 days with MeOH (5.0 L). The MeOH extract was concentrated under reduced pressure at 37 °C to obtain MeOH extract (120 g). The MeOH extract (fraction A, 120 g) was purified by column chromatography (CC) (5.4 kg of reversed-phase C18 silica gel, 200–400 mesh; H_2_O/MeOH 90:1–0:1, 1800 mL) to afford 12 fractions: A1–A12. Fraction A2 (6.8 g) was subjected to CC (305 g of C18 silica gel, 230–400 mesh (40–63 μm); H_2_O/MeOH 19:1–1:1, 900 mL fractions) to give 11 subfractions: A2-1–A2-11. Fraction A2-3 (335 mg) was purified by HPLC (ODS column, H_2_O/MeOH 1:1, 2.0 mL min^−1^) to obtain rutin (31.3 mg) (*t*_R_ 2.8 min).

Fraction A3 (5.6 g) was chromatographed on C18 silica gel (230–400 mesh, 250 g), eluting with H_2_O/MeOH (9:1–3:7) to give 8 fractions (each 700 mL, A3-1–A3-9). Fraction A3-6 (347 mg) was purified by HPLC (ODS column, H_2_O/acetonitrile, 84:16, 1.0 mL min^−1^) to afford hyperoside (41.6 mg) (*t*_R_ 9.0 min) and isoquercetin (25.4 mg) (*t*_R_ 9.5 min). Fraction A5 (5.3 g) was chromatographed on C18 silica gel (230–400 mesh, 240 g) and eluted with H_2_O/MeOH (7:3–2:8) to give 10 fractions (each 650 mL, A5-1–A5-10). Fraction A5-6 (326 mg) was purified by HPLC (ODS column, H_2_O/MeOH, 3:1, 1.0 mL min^−1^) to afford scopoletin (26.2 mg) (*t*_R_ 12.8 min). Fraction A8 (6.8 g) was separated by column chromatography over Sephadex LH-20 and eluted with 100% MeOH to yield 8 fractions (A8-1–A8-8). Fraction A8-3 (530 mg) was purified by HPLC (ODS column, H_2_O/MeOH, 19:1, 1.0 mL min^−1^) to afford 5-HMF (32.3 mg) (*t*_R_ 3.2 min). The structures of rutin, hyperoside, isoquercetin, scopoletin and 5-HMF were identified by nuclear magnetic resonance (NMR) spectra acquired using a Bruker Avance 600 MHz spectrometer (Bruker, Bremen, Germany) (Figures S1–S5).

### 2.4. Reverse-Phase HPLC

The reversed-phase HPLC assay for the measurement of the seven components was performed as previously described, with minor modifications [28,29]. Reversed-phase separations were executed using a LiChrospher 100 RP-18 Endcapped (5 μm; column of dimensions 4.6 × 250 mm). A reversed-phase HPLC separation was conducted using a mobile phase of 0.2% acetic acid in water (*v*/*v*) (solvent A) and acetonitrile (solvent B) as follows: 0–8 min, isocratic elution with 95% A; 8–15 min, isocratic elution with 8% B; 15–35 min, isocratic elution with 10% B; 35–45 min, isocratic elution with 12% B; 45–55 min, isocratic elution with 15% B; 55–65 min, isocratic elution with 25% B; 65–75 min, isocratic elution with 45% B; 75–95 min, isocratic elution with 55% B; 95–105 min, isocratic elution with 65% B; 105–130 min, gradient elution from 65 to 100% B; 130–140 min, back to initial 95% A. The flow rate was 1.0 mL/min, and the injection volume was 500 μL at room temperature. Peaks were detected at 280 nm. Different compounds were identified by retention time.

### 2.5. Measurement of Total Phenolic Content (TPC)

The TPC of different solvent extracts was measured using a previously reported method [30].

### 2.6. Measurement of Total Flavonoid Content (TFC)

The TFC of different solvent extracts was determined using a previously reported method [31].

### 2.7. DPPH Radical Scavenging Assay

This assay was determined using the procedure previously reported [32].

### 2.8. ABTS Radical Scavenging Assay

This assay was determined using the procedure previously reported [33].

### 2.9. Superoxide Radical Scavenging Assay

This assay was determined using the method previously reported [32].

### 2.10. Ferric Reducing Antioxidant Power (FRAP) Assay

The FRAP assay was determined using the method previously reported [34].

### 2.11. α-Glucosidase Inhibitory Assay

This assay was performed according to the previous method [35].

### 2.12. Acetylcholinesterase Inhibitory Assay

The AChE inhibitory assay was performed according to the previous method, with slight modifications [36]. First, 1 mL of DTNB (15 mM) solution, 1 mL of AChI (15 mM) solution, 1 mL of AChE (0.2 U/mL), and various concentrations of PS (50–400 μg/mL) were prepared using sodium phosphate buffer (0.1 M, pH 8.0). The reaction was started by adding 140 μL PBS, 10 μL DTNB solution, 15 μL AChE solution and 20 μL sample. The reaction mixture was incubated at 20 °C in the dark for 10 min, and 10 μL of AChI solution was added. Finally, the reaction mixture was incubated at 20 °C for 10 min, and the absorbance was measured at 405 nm.

### 2.13. Cell Culture

Murine RAW264.7 macrophages were cultured in DMEM medium containing 10% fetal bovine serum (FBS) and 1% penicillin at 37 °C, 5% CO_2_ [37].

### 2.14. Nitric Oxide Inhibitory Assay

The NO inhibitory assay was performed based on the reference method [37].

### 2.15. MTT Assay

The MTT assay was carried out according to the previous method [37].

### 2.16. Western Blot Analysis

The Western blot analysis was performed using the reference method with slight modifications [37]. RAW264.7 cells were seeded in 6 cm dishes for 24 h. The cells were treated with sample (12.5, 25, and 50 µM) and LPS (100 ng/mL). After treatment with drugs, cells were washed with cold PBS, and proteins were collected with lysis buffer containing protein inhibitors. After quantification, equal amounts of protein samples were separated using 5–8% SDS-polyacrylamide gels. The proteins were transferred to the membrane by electrophoresis. The membranes were soaked in 2% BSA blocking buffer for 2 h and then washed 2–3 times with TBST. Next, the membranes were visualized with primary antibody (iNOS, TNF-α, and IL-6) and soaked for 1 day. The next day, membranes were washed again with TBST 2–3 times and then soaked with secondary antibody for 2 h. Finally, immuno-reactivity was detected with ECL reagents, exposed with a luminometer photometer, and quantified with Image J.

### 2.17. Molecular Modeling Docking Study

The in silico evaluation was performed with AutoDock Vina (ADT ver. 4.0.1) software [38]. Crystal structures of AChE were retrieved from the Protein Data Bank (PDB: 1C2B), and hydrogen atoms were added to prepare the docked receptors. The 3D structures of the ligands were constructed in the Chem3D program. The addition of hydrogen, the Gasteiger charge measurement of the protein atoms and the selection of the flexible twist of the ligand were performed by AutodockTools (ADT ver. 1.5.6). The grid dimensions were designed as 15 Å × 15 Å × 15 Å for isoquercetin, 14 Å × 14 Å × 14 Å for scopoletin, 10 Å × 10 Å × 10 Å for ru-tin, and 20 Å × 20 Å × 20 Å for chlorogenic acid. Finally, the crystal structure of iNOS was retrieved from the Protein Data Bank (PDB: 1M9T). The grid dimensions were designed as 17Å × 17Å × 17Å for rutin, 15Å × 15Å × 16Å for isoquercetin, 20Å × 16Å × 14Å for hyperoside, and 16Å × 16Å × 16Å for quercetin. The binding affinity energy was provided as a docking fraction and measured in kcal/mol. The best interactions were only considered to be the highest fraction of the gestalt. The visualization of the best docking interactions was analyzed in Biovia Discovery Studio Client 2021 [39].

### 2.18. Statistical Analysis

Statistical analysis was carried out using the t-test, and all assays were performed at least three times and expressed as mean ± standard error of the mean (SEM). Less than 0.05 was considered statisti-cally significant.

## 3. Results and Discussion

### 3.1. Measurement of TPC, TFC and Yield in Various Solvent Extracts

We studied the TPC, TFC and yields in different solvent extracts of *Polygonatum sibiricum* (PS) and processed *Polygonatum sibiricum* (PPS). Table 1 displays TPC, TFC, and extraction yield of CH_2_Cl_2_, EtOAc, acetone, MeOH, EtOH, and water extracts from PS and PPS. The yields of various solvent extracts were ranged from 13.49 ± 1.53% (methanol extract) to 0.35 ± 0.12% (dichloromethane extract) of PS, and 15.90 ± 2.64% (water extract) to 1.25 ± 0.03% (dichloromethane extract) of PPS. The dichloromethane extract of PS and ethyl acetate extract of PPS showed the highest TPC with 77.50 ± 7.34 and 60.47 ± 1.91 mg/g, respectively. The highest TFC (86.02 ± 1.54 and 98.30 ± 0.47 mg/g) were found in the ethyl acetate extracts of PS and PPS, respectively, among all solvent extracts (Table 1).

### 3.2. DPPH Free-Radical Scavenging Effect of Various Solvent Extracts

The DPPH radical scavenging ability of different solvent extracts is displayed in Table 2. BHT was used as a positive control. From the results, the dichloromethane (SC_50_ = 236.14 ± 3.89 μg/mL) and acetone extracts (SC_50_ = 278.31 ± 3.26 μg/mL) of PS showed relatively strong antioxidant effects by DPPH radical scavenging assay among all extracts.

### 3.3. ABTS Free-Radical Scavenging Effect of Various Solvent Extracts

The ABTS radical scavenging ability of different solvent extracts is displayed in Table 2. The acetone extract (SC_50_ = 229.37 ± 5.59 μg/mL) of PS showed the greatest ABTS radical scavenging effect, followed by dichloromethane extract (SC_50_ = 240.49 ± 5.68 μg/mL), ethanol extract (SC_50_ = 245.48 ± 3.59 μg/mL), and methanol extract (SC_50_ = 364.48 ± 3.21 μg/mL).

### 3.4. Superoxide Radical Scavenging Effect of Various Solvent Extracts

The results are shown in Table 2. All extracts had no scavenging activity against superoxide radicals (SC_50_ > 400 g/mL), except for the EtOAc extract (SC_50_ = 190.23 ± 1.09 μg/mL) and water extract (SC_50_ = 294.54 ± 7.28 μg/mL) of PS.

### 3.5. Ferric Reducing Antioxidant Power (FRAP) Effect of Various Solvent Extracts

The FRAP activities of all extracts are shown in Table 2, and BHT was used as a positive control. The FRAP assay is expressed as millimoles (mM) of Trolox equivalents (TE) per gram of extract. The dichloromethane extract (667.08 ± 18.56 mM TE/g), ethyl acetate extract (651.03 ± 20.56 mM TE/g) and acetone extract (515.84 ± 24.86 mM TE/g) of PS and ethyl acetate extract (604.38 ± 3.73 mM TE/g) of PPS showed relatively high antioxidant capacity. Based on the aforementioned antioxidant data (DPPH and FRAP assays), the dichloromethane extracts of PS showed relatively high antioxidant effects among all the extracts. As for the ethyl acetate extract of PS, it showed a higher antioxidant effect by the superoxide radical scavenging test.

### 3.6. Anti-α-Glucosidase Effect of Various Solvent Extracts

As presented in Table 3, the ethyl acetate extract of *P. sibiricum* had the strongest anti-α-glucosidase activity (IC_50_ = 22.34 ± 1.66 μg/mL), followed by acetone extract (IC_50_ = 26.13 ± 2.48 μg/mL) and dichloromethane extract (IC_50_ = 34.29 ± 6.26 μg/mL).

The dichloromethane, ethyl acetate and acetone extracts of PS were more effective than the positive control, acarbose (IC_50_ = 379.07 ± 4.23 μg/mL). These results suggest that the low-polarity solvent extracts of PS had a higher α-glucosidase inhibitory effect (Table 3).

### 3.7. Acetylcholinesterase (AChE) Inhibitory Effect of Various Solvent Extracts

Alzheimer’s disease (AD) is a progressive, neurodegenerative disease characterized by a decline in cognitive and memory functions. Acetylcholinesterase (AChE) inhibitors block the AChE enzyme, thus revitalizing cholinergic action to improve memory and cognitive function. The AChE inhibitory effect of each extract is presented in Table 3, and the AChE inhibitor, chlorogenic acid, is used as a positive control [40]. From our test results, the dichloromethane extract (IC_50_ = 60.90 ± 6.18 μg/mL) of PS and the ethyl acetate extract (IC_50_ = 32.60 ± 5.27 μg/mL) and dichloromethane extract (IC_50_ = 56.27 ± 7.11 μg/mL) of PPS showed the most effective AChE-inhibitory effect among all extracts.

### 3.8. Nitric Oxide Inhibitory (NO) Effect of Various Solvent Extracts

**Figure 2:**
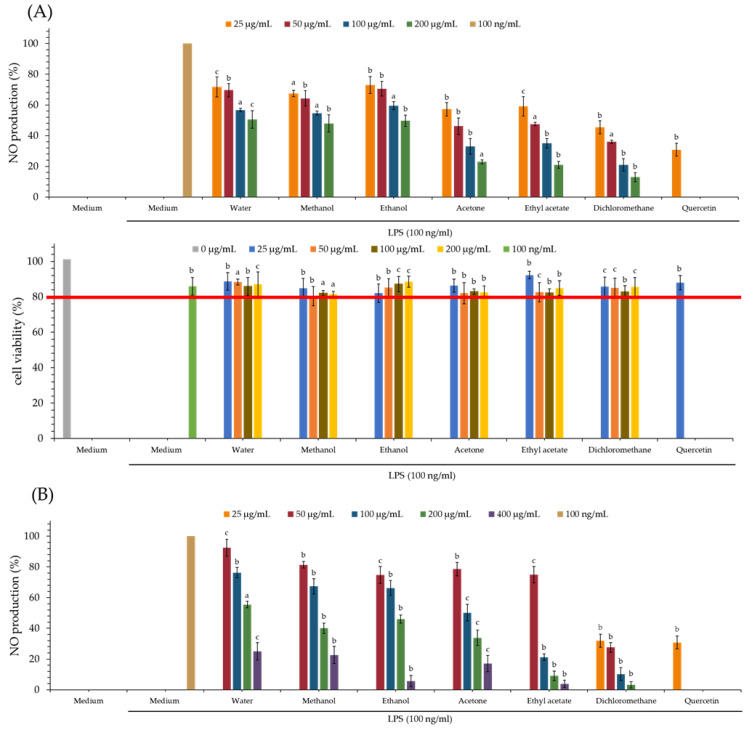
Nitric oxide inhibitory and MTT assays of various solvent extracts of PS (**A**) and PPS (**B**). The data are displayed as mean ± SD (*n* = 3); Quercetin is used as a positive control; PS means *Polygonatum sibiricum*. PPS means processed *Polygonatum sibiricum*. ^a^ *p* < 0.001, ^b^ *p* < 0.01, and ^c^ *p* < 0.05 compared to control.

The NO inhibitory assay of each extract is displayed in Table 4 and Figure 2. Quercetin is used as a positive control. From our test results, the dichloromethane extract (IC_50_ = 18.84 ± 1.80 μg/mL) of PS and dichloromethane extract (IC_50_ = 27.48 ± 6.99 μg/mL) of PPS show the greatest NO inhibitory effect among all solvent extracts.

### 3.9. MTT Assay of Various Solvent Extracts

The MTT assay is mainly used to detect the cytotoxicity of tested samples. From the MTT assay (Figure 2), the cell survival rate of various extracts of PS and PPS is higher than 80%, and this suggests that the inhibitory activity of these extracts against LPS-induced NO production does not result from their cytotoxicities.

### 3.10. Quantitation of Bioactive Components in Various Solvent Extracts

**Figure 3:**
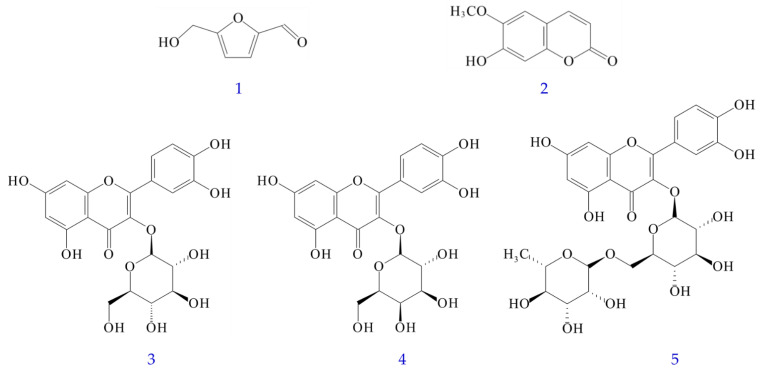
Chemical structures of 5-hydroxymethylfurfural (**1**), scopoletin (**2**), isoquercetin (**3**), hyperoside (**4**), and rutin (**5**) from *Polygonatum sibiricum*.

Figures S1–S18 showed the quantification of bioactive compounds in various solvent extracts of *Polygonatum sibiricum* by reversed-phase HPLC analysis and the ^1^H-NMR spectra of bioactive compounds. Table S1 displayed retention time, limit of detection (LOD), limit of quantification (LOQ), and regression analysis for five components of *P. sibiricum* in reversed phase HPLC. The amounts of the five bioactive compounds in different solvent extracts are illustrated in Table 5. The content of five bioactive compounds in different solvent extracts of PS ranged from the highest value of 17.25 ± 1.68 mg/g (dichloromethane extract) to the lowest value of 6.77 ± 0.50 mg/g (water extract), in order of dichloromethane > ethanol > ethyl acetate > methanol > acetone > water extract. In addition, the sum of the five bioactive compounds in different solvent extracts of PPS ranged from a maximum value of 27.49 ± 2.31 mg/g (dichloromethane extract) to a minimum value of 17.19 ± 2.66 mg/g (water extract), in order of dichloromethane > acetone > ethanol > methanol > ethyl acetate > water extract. Both PS and PPS showed the presence of 5-HMF in different solvent extracts, but the content of PPS was much higher than that of PS. However, scopoletin was only present in PS and not in PPS (Table 5). The five major bioactive compounds are 5-hydroxymethylfurfural (5-HMF) (**1**), scopoletin (**2**), isoquercetin (**3**), hyperoside (**4**), and rutin (**5**) (Figure 3).

### 3.11. Antioxidant Effects of Isolated Components

Antioxidant assay results of isolated components (**1**–**5**) are displayed in Table 6. Rutin (**5**) (SC_50_ = 5.60 ± 0.34 μM) showed the strongest DPPH radical scavenging effect, followed by hyperoside (**4**) (SC_50_ = 12.46 ± 4.02 μM), and isoquercetin (**3**) (SC_50_ = 12.64 ± 3.21 μM). Rutin (**5**) (SC_50_ = 15.43 ± 0.25 μM) also showed the most potent ABTS scavenging effect, followed by isoquercetin (**3**) (SC_50_ = 22.73 ± 1.17 μM) and hyperoside (**4**) (SC_50_ = 29.26 ± 0.51 μM). Compounds **3**, **4** and **5** displayed higher superoxide radical scavenging effects than **1** and **2**. In addition, compounds **3** and **4** showed a more effective antioxidant effect than BHT by FRAP assay (Table 6).

### 3.12. Anti-α-Glucosidase Effects of Isolated Components

As displayed in Table 7, scopoletin (**2**) (IC_50_ = 23.63 ± 7.22 μM) showed the most effective anti-α-glucosidase effect, followed by isoquercetin (**3**) (IC_50_ = 159.73 ± 3.12 μM), hyperoside (**4**) (IC_50_ = 208.14 ± 5.70 μM), and rutin (**5**) (IC_50_ = 331.15 ± 3.81 μM). Compounds **2**–**5** showed more potent anti-α-glucosidase effect than the positive control, acarbose (IC_50_ = 550.15 ± 7.65 μM) (Table 7).

### 3.13. Acetylcholinesterase (AChE) Inhibitory Effects of Isolated Compounds

As shown in Table 7, isoquercetin (**3**) (IC_50_ = 23.13 ± 3.15 μM) exhibited the strongest anti-AChE effect, followed by scopoletin (IC_50_ = 32.35 ± 2.05 μM), rutin (**5**) (IC_50_ = 33.09 ± 5.43 μM), 5-HMF (IC_50_ = 81.46 ± 11.05 μM), and hyperoside (**4**) (IC_50_ = 121.10 ± 10.70 μM). Compounds **2**, **3** and **5** showed more effective anti-AChE activities than the positive control, chlorogenic acid (68.23 ± 2.90 μM).

### 3.14. Nitric Oxide (NO) Inhibitory Effect of Isolated Components

**Figure 4:**
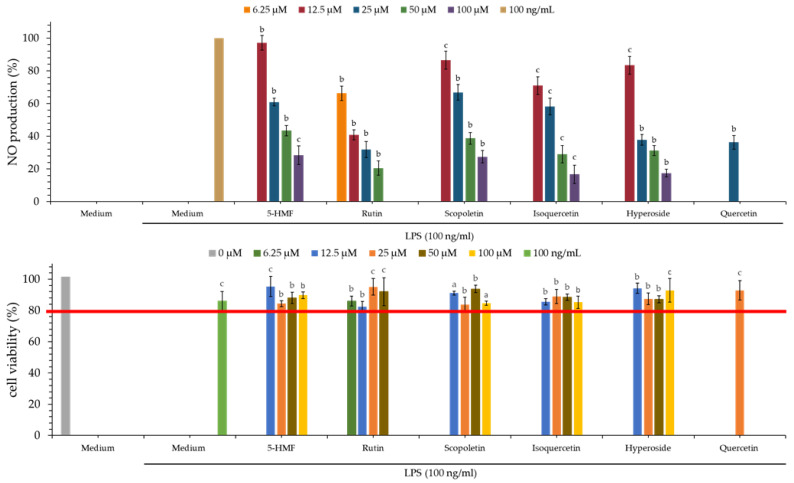
Nitric oxide inhibitory and MTT assays of isolated compounds. The data are displayed as mean ± SD (*n* = 3). Quercetin is used as a positive control; ^a^ *p* < 0.001, ^b^ *p* < 0.01, and ^c^ *p* < 0.05 compared to control.

As showed in Table 8 and Figure 4, rutin (**5**) (IC_50_ = 9.89 ± 1.36 μM) showed the strongest nitric oxide inhibitory effect, followed by isoquercetin (**3**) (IC_50_ = 17.03 ± 1.28 μM), hyperoside (**4**) (IC_50_ = 18.87 ± 1.68 μM), 5-HMF (**1**) (IC_50_ = 34.90 ± 8.80 μM) and scopoletin (**2**) (IC_50_ = 36.26 ± 4.65 μM). Compounds **3** and **5** exhibited more effective anti-NO activities than the positive control, quercetin (18.26 ± 0.54 μM).

### 3.15. MTT Assay of Isolated Components

As shown in Figure 4, the cell survival rate of all isolated compounds at 100 μM was higher than 80%, and this suggested that the inhibitory activity of these isolated compounds against LPS-induced NO production does not result from their cytotoxicities.

### 3.16. Western Blot Analysis of Isolated Components

**Figure 5:**
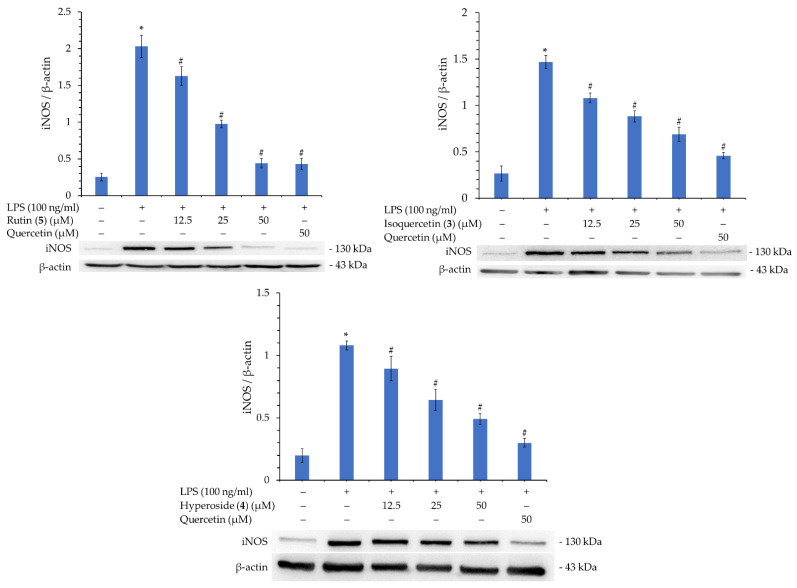
Western blot analysis of iNOS protein of rutin (**5**), isoquercetin (**3**), and hyperoside (**4**). Quantification data of iNOS/β-actin are expressed as mean ± SEM. Quercetin was used as positive control. * *p* < 0.05 vs. control group, ^#^ *p* < 0.05 vs. LPS group.

Rutin (**5**), isoquercetin (**3**), and hyperoside (**4**), with their better inhibitory activity against NO production, were selected for further analysis of their inhibitory effect on iNOS. As shown in Figure 5, compounds **3**, **4**, and **5** significantly inhibited iNOS production in a concentration-dependent manner. Therefore, the reason for their inhibition of NO production can be verified.

**Figure 6:**
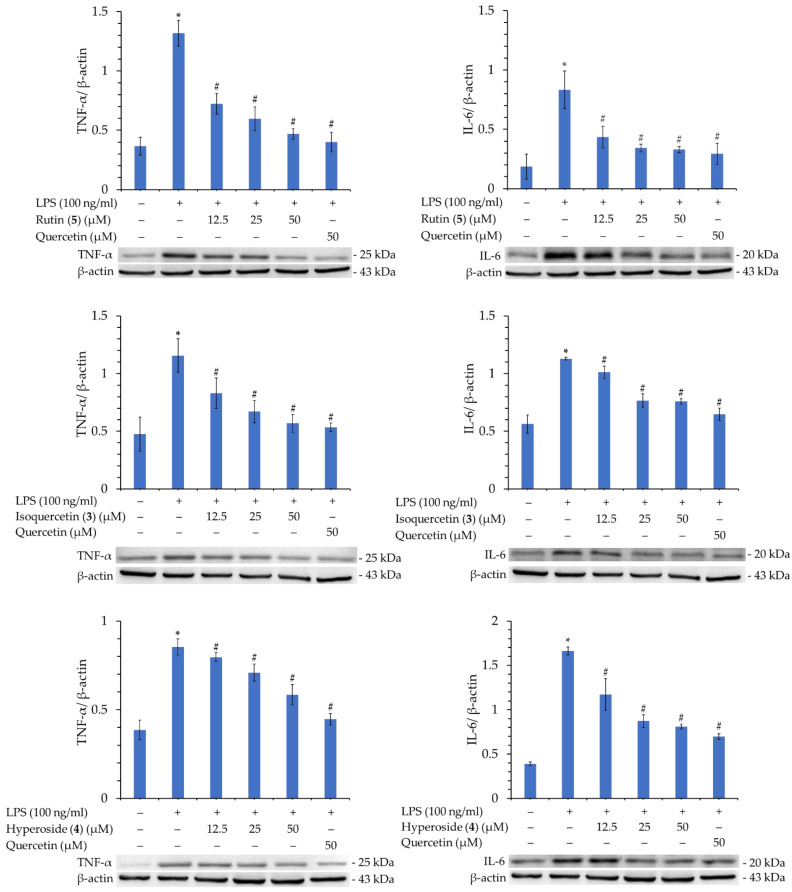
Western blot analysis of TNF-α and IL-6 proteins of rutin (**2**), isoquercetin (**3**), and hyperoside (**5**). Quantification data of TNF-α/β-actin and IL-6/β-actin are expressed as mean ± SEM; Quercetin was applied as positive control. * *p* < 0.05 vs. control group, ^#^ *p* < 0.05 vs. LPS group.

In addition, compounds **3**, **4**, and **5** were further tested for their activity in inhibiting the production of TNF-α and IL-6. As displayed in Figure 6, compounds **3**, **4**, and **5** also significantly inhibited the production of TNF-α and IL-6 in a concentration-dependent manner. This verifies that these compounds possess potent anti-inflammatory activity.

### 3.17. Molecular Docking Study

To further investigate the interaction between biologically active compounds and acetylcholinesterase and try to explain how these compounds exert their antagonistic effects, docking models of compounds were generated using the Discovery Studio 2021 (Accelrys, San Diego, CA, USA) modeling program. The crystal structure (PDB: 1C2B) of acetylcholinesterase from *Electrophorus electricus* was also used in this study [41].

The molecular docking simulations were performed along with the binding affinity calculations for PDB: 1C2B (acetylcholinesterase) and active compounds. The interactions between active compounds and PDB: 1C2B were displayed in the best-docked poses for the calculations. These results demonstrate the high accuracy of the existing simulation system, thereby supporting further calculations. The lowest binding energy for each ligand was considered the optimal conformation, and the binding affinities are shown in Table 9. In this study, chlorogenic acid was used as a positive control. The binding energies of isoquercetin (**3**) (−7.5 kcal/mol), scopoletin (**2**) (−7.0 kcal/mol), and rutin (**5**) (−6.8 kcal/mol) were lower than those of chlorogenic acid (−5.3 kcal/mol), suggesting that these compounds can have better docking ability with the crystal structure of PDB: 1C2B.

**Figure 7:**
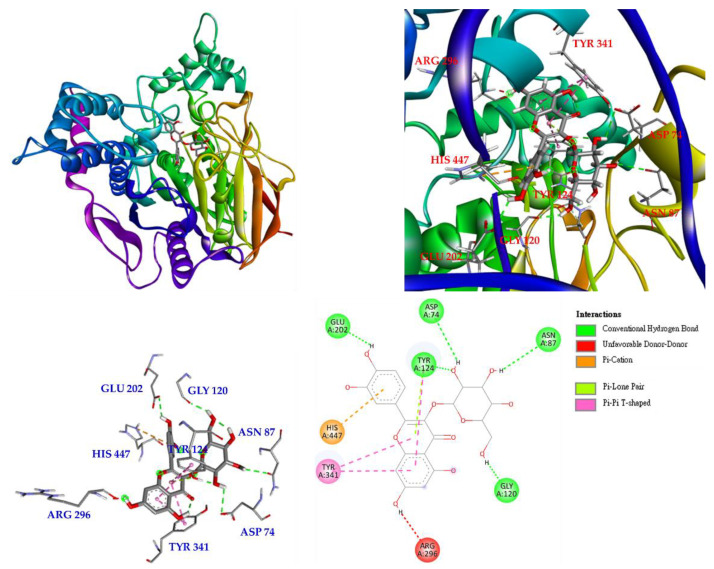
Interactions of isoquercetin (**3**) with active sites of *E. electricus* AChE.

Interactions of isoquercetin (**3**) with the active sites of *E. electricus* acetylcholinesterase (AChE) are shown in Figure 7. Isoquercetin is bound to GLU 202, TYR 124, ASP 74, ASN 87 and GLY 120 via conventional hydrogen bonding, with unfavorable donor-donor interactions found on ARG 296. There are other interactions with isoquercetin, such as π-cation, π-lone pair and π-π T-shaped interactions, which can mainly form stable complexes of isoquercetin and protein.

**Figure 8:**
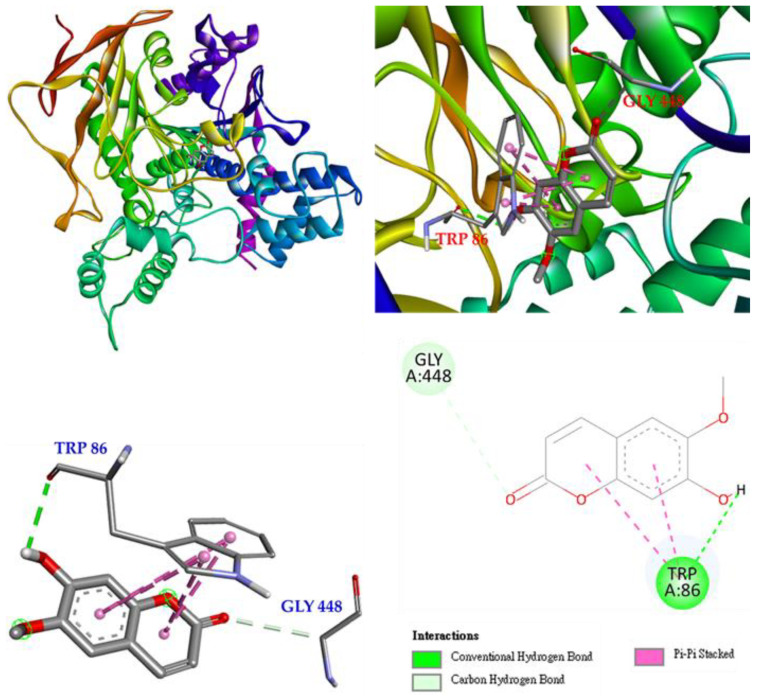
Interactions of scopoletin (**2**) with active sites of *E. electricus* AChE.

Molecular docking of scoloptein (**2**) and *E. electricus* acetylcholinesterase is shown in Figure 8. Scoloptein binds to GLY 448 via carbon-hydrogen bonding and to TRP 86 via conventional hydrogen bonding. In addition, there is a π-π stacked interaction with scoloptein to make a stable complex of scoloptein and AChE.

Interactions of rutin (**5**) with the active sites of *E. electricus* AChE are shown in Figure S19. Rutin (**5**) binds to TYR 124 and GLN 71 via conventional hydrogen bonding and to TRP 286, PRO 88, and SER 125 via carbon-hydrogen bonding. Unfavorable donor-donor and unfavorable acceptor-acceptor interactions were found on PHE 295, TYR 341, and TYR 337. There are other interactions with rutin, such as π-π T-shaped, alkyl, and π-π stacked interactions to make a stable complex of rutin and AChE.

**Figure 9:**
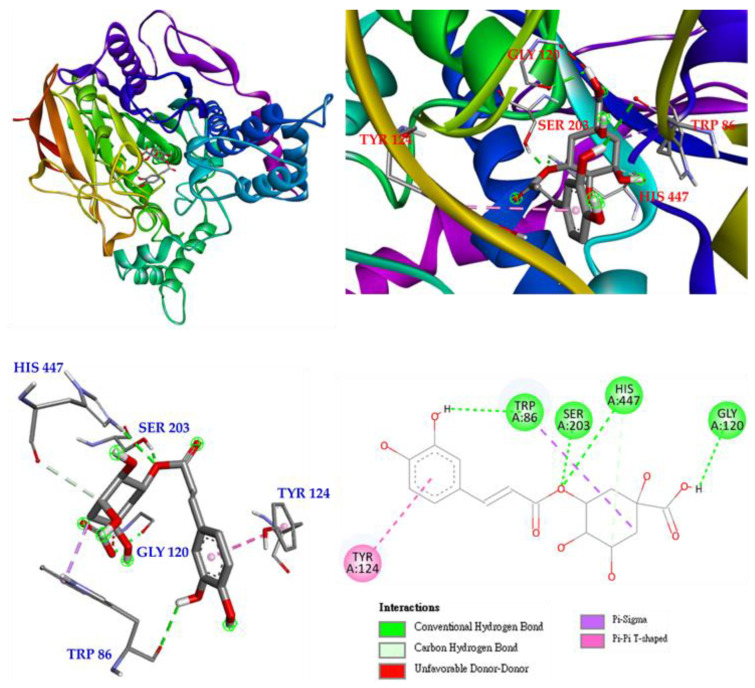
Interactions of chlorogenic acid with active sites of *E. electricus* AChE.

From the above results, it can be concluded that the binding energies of compounds **2**, **3**, and **5** are better than that of chlorogenic acid, as shown in Table 9. Likewise, the anti-AChE effects of compounds **2**, **3**, and **5** were confirmed to be superior to that of chlorogenic acid (Figure 9).

According to the results of the NO production inhibition test (Table 8) and the Western blotting results of a related protein, inducible nitric oxide synthase (iNOS) (Figure 6), the three active compounds, rutin (**5**), isoquercetin (**3**), and hyperoside (**4**), all have anti-inflammatory potential. Therefore, these three compounds were used in a molecular docking model to see their binding abilities to iNOS. The 3D structure (PDB: 1M9T) of iNOS used as a docking model is from *Mus musculus*, and the active site consists of four pockets, where the substrate-binding S pocket contains heme [42].

**Figure 10:**
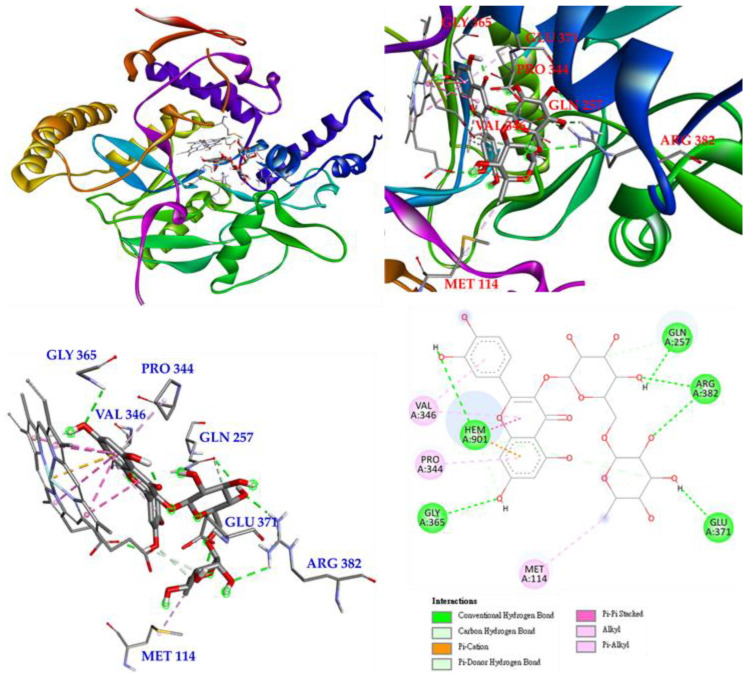
Interactions of rutin (**5**) with active sites of *M. musculus* iNOS.

Interactions of rutin (**5**) with the active sites of *Mus musculus* iNOS are shown in Figure 10. Rutin mainly forms a conventional hydrogen bond with GLY 365, and even with GLN 257, ARG 382 and GLU 371, and it also forms π-alkyl interactions with VAL 346 and PRO 344. Rutin forms multiple interactions with HEM 901, such as π-cations, π-π stacked, π-donor hydrogen bonds, carbon-hydrogen bonds, and conventional hydrogen bonds, which increases the parallelism between rutin and HEM 901 and increases the affinity of rutin and iNOS.

**Figure 11:**
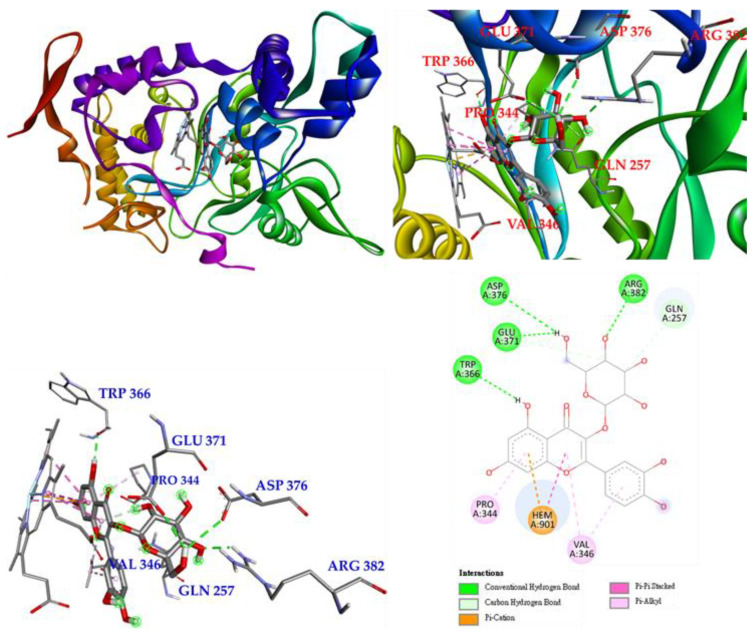
Interactions of isoquercetin (**3**) with active sites of *M. musculus* iNOS.

The next compound is isoquercetin (**3**), which mainly forms conventional hydrogen bonds with TRP 366 and even with GLU 371, ASP 376 and ARG 382, and also forms π-alkyl interactions with PRO 344 and VAL 346. Carbon hydrogen bonds are observed between isoquercetin and GLN 371 and GLN 257. Most importantly, isoquercetin forms π-π-carbon and π-π stacked interactions with HEM 901 to remain parallel to HEM 901 and increase the affinity of isoquercetin and iNOS. (Figure 11).

**Figure 12:**
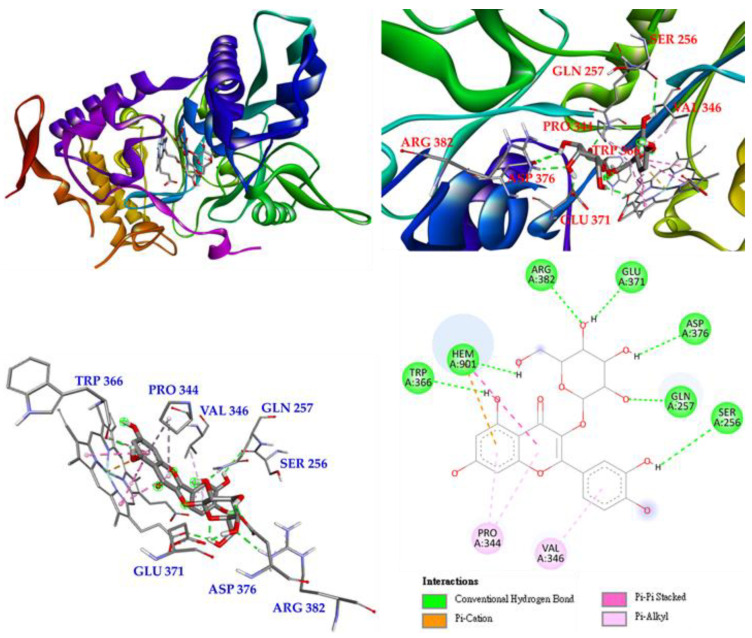
Interactions of hyperoside (**4**) with active sites of *M. musculus* iNOS.

The last is hyperoside (**4**), which mainly forms conventional hydrogen bonds with TRP 366 as well as with GLU 371, ASP 376, GLN 257, SER 256 and ARG 382. Hyperoside also forms π-alkyl interactions with PRO 344 and VAL 346. In addition, hyperoside forms π-cation, π-π stacked, and conventional hydrogen bond interactions with HEM 901 to increase their affinity (Figure 12).

**Figure 13:**
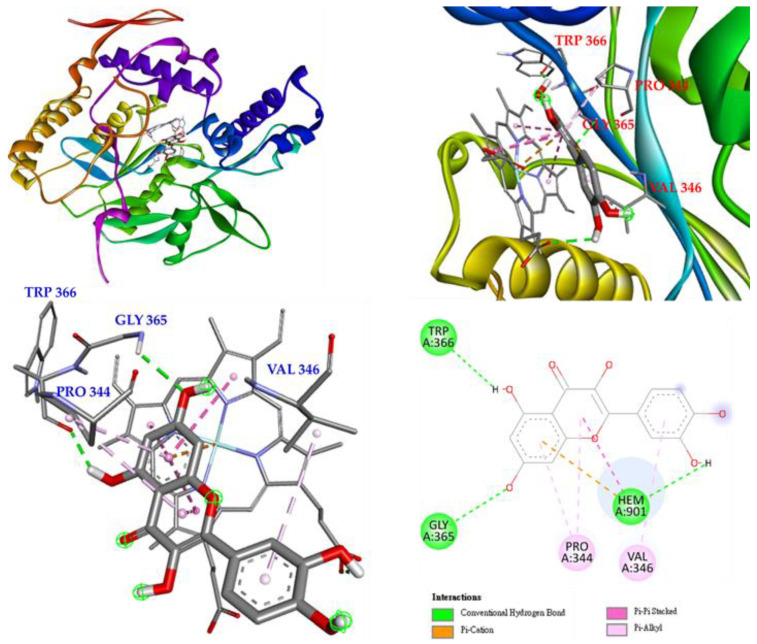
Interactions of quercetin with active sites of *M. musculus* iNOS.

According to the binding energy data presented in Table 10, the binding affinity of rutin (**5**) was significantly higher than compounds **1**–**4** and the positive control quercetin (Figure 13). This indicated that rutin (**5**) has the greatest binding affinity with iNOS, which is consistent with the strongest anti-NO activity of rutin (**5**) presented in Table 8.

## 4. Discussion

Various approaches have been devoted to extracting natural products (i.e., herbs, plants and fungi) to replace modern medicines. For many years, the most common extraction methods were boiling or decoction, both of which were considered cost-effective and easy to perform. Researching and finding natural ingredients with health benefits from medicinal plants is a hot trend these days. In recent studies, organic solvents have been used to obtain extracts of natural products, including various metabolites, depending on the polarity and properties of these specific compounds [43]. Other exogenous factors may also affect natural product extraction, including the type of solvent used, the temperature altered during extraction, and the property of the plant material [44]. Changes in solvent polarity can lead to differences in phytochemical composition and biological activity. Therefore, the rhizomes of *Polygonum sibiricum* were extracted using solvents of different polarities, and the biological activities of the extracts and isolated compounds were evaluated. The results show that by using solvents of different polarities, different extracts and components with different contents can be obtained and show different biological activities.

The ABTS and DPPH assays are mainly used to evaluate the antioxidant effects of natural compounds, which are usually related to the proton radical scavenging or hydrogen donating capacity of the compounds [45]. The superoxide radical scavenging effect is assessed by superoxide anion derived from dissolved oxygen by phenazine methosulphate/nicotinamide adenine dinucleotide (PMS/NADH) coupling reaction, which reduces nitroblue tetrazolium (NBT) [46]. The FRAP assay is evaluated by the antioxidant potential of various extracts and samples by reducing iron (Fe^3+^) complexes to ferrous (Fe^2+^) complexes [47]. In our study, dichloromethane and acetone extracts of PS show higher antioxidant effects than other extracts in DPPH and ABTS assays, which correlates with their higher TPC than other extracts.

A comparative assessment of the TPC, TFC, and antioxidant effects (DPPH, ABTS, superoxide, and FRAP) of various solvent extracts (CH_2_Cl_2_, EtOAc, acetone, EtOH, MeOH, and water) of PS and PPS was demonstrated for the first time in this study. This may give a direction for the choice of suitable solvents for TFC, TPC, and antioxidant extraction applications. According to the antioxidant assay result, rutin (**5**) (SC_50_ = 5.60 ± 0.34 μM) showed the strongest DPPH radical scavenging effect. Compounds **3**, **4** and **5** displayed higher superoxide radical scavenging effects than **1** and **2**. In addition, compounds **3** and **4** showed a more effective antioxidant effect than BHT by FRAP assay.

Among all antidiabetic drugs used to treat type 2 diabetes, α-glucosidase inhibitors are considered to be the most effective drugs for reducing postprandial hyperglycemia. Currently, acarbose, voglibose, and miglitol are effective enzyme inhibitors for the treatment of postprandial hyperglycemia, but these inhibitors have side effects on the gastrointestinal tract; therefore, they are not suitable for long-term use [48]. Scopoletin (**2**) (IC_50_ = 23.63 ± 7.22 μM), isoquercetin (**3**) (IC_50_ = 159.73 ± 3.12 μM) and hyperoside (**4**) (IC_50_ = 208.14 ± 5.70 μM) showed more potent anti-α-glucosidase activity than acarbose (IC_50_ = 550.15 ± 7.65 μM). Compound **2** is about 10 times more potent than acarbose against α-glucosidase.

Alzheimer’s disease (AD) is a progressive degenerative brain disease in which cognitive and memory functions deteriorate. It is primarily treated by AChE inhibitors that increase the cholinergic effects of the brain. In contrast, chlorogenic acid, a phenolic acid derived from food (fruits and vegetables), has been shown to have neuroprotective properties associated with Alzheimer’s disease [24,49]. From the previous research data, isoquercetin (**3**) has attracted the attention of many researchers because of its potential to improve brain memory function through different mechanisms, so it is believed to have a certain role in delaying the progression of AD memory loss [50]. In our study, isoquercetin (**3**) (IC_50_ = 23.13 ± 3.15 μM), scopoletin (**2**) (IC_50_ = 32.35 ± 2.05 μM) and rutin (**5**) (IC_50_ = 33.09 ± 5.43 μM) also show potent anti-AChE effects and deserve further investigation.

Based on the results of the anti-AChE assay, compound **3** exhibited the most significant inhibitory effect among all isolated compounds; therefore, the interaction between AChE and **3** was assessed by molecular docking. Compound **3** showed the best binding energy (−7.5 kcal/mol) compared to chlorogenic acid (−5.3 kcal/mol). This revealed that **3** could dock into the pocket of the crystal structure of AChE from *E. electricus* more efficiently than chlorogenic acid.

The inflammatory response is a defense mechanism of the body. A general inflammatory response leads to tissue protection and regeneration after injury and, in this way, responds to injury and infection. Under inflammatory conditions, activated macrophages can exhibit detrimental effects involving the overproduction of inflammatory cytokines such as NO, TNF-α and IL-6. Therefore, inhibiting the aberrant activation of macrophages may have therapeutic potential in the treatment of inflammation-related degenerative diseases. In our study, compounds **3** (IC_50_ = 17.03 ± 1.28 μM) and **5** (IC_50_ = 9.89 ± 1.36 μM) exhibited more effective anti-NO activities than the positive control, *quercetin* (IC_50_ = 18.26 ± 0.54 μM). Compounds **3**, **4**, and **5** also significantly inhibited the production of iNOS, TNF-α and IL-6 in a concentration-dependent manner.

Based on Western blot analysis of iNOS expression, compound **5** showed the strongest inhibitory effect among all isolated compounds. Therefore, the interaction between iNOS and compound **5** was assessed by molecular docking. Compound **5** showed the best binding energy (−9.5 kcal/mol) compared to quercetin (−7.9 kcal/mol). This revealed that **5** could dock into the pocket of the crystal structure of iNOS from *M. musculus* more efficiently than quercetin. In addition, our study performed molecular docking for the first time to calculate the binding energy of **5** with *M. musculus* iNOS.

## 5. Conclusions

Different solvent extracts of *P. sibiricum* (PS) and processed *P. sibiricum* (PPS) were investigated with anti-AChE, anti-α-glucosidase, anti-inflammatory and antioxidant assays. In this study, the CH_2_Cl_2_ extract of PS revealed the strongest antioxidant activity among all solvent extracts by ABTS, DPPH and FRAP assays. The EtOAc extract (IC_50_ = 22.34 ± 1.66 μg/mL) of PS displayed the highest anti-α-glucosidase effect compared with all other solvent extracts. The CH_2_Cl_2_ extract (IC_50_ = 18.84 ± 1.80 μg/mL) of PS showed the strongest anti-NO effect. The EtOAc extract (IC_50_ = 32.60 ± 5.27 μg/mL) of PPS revealed the most potent anti-AChE activity. Five isolated components from *P. sibiricum* were quantified by HPLC and identified as 5-hydroxymethylfurfural (**1**), scopoletin (**2**), isoquercetin (**3**), hyperoside (**4**), and rutin (**5**). Furthermore, a comparative assessment for the quantification of these major active compounds (**1**–**5**) of various solvent extracts (CH_2_Cl_2_, acetone, water, MeOH, EtOH, and EtOAc) from *P. sibiricum* (PS) and processed *P. sibiricum* (PPS) by HPLC analyses was first carried out in this research.

Bioactivity assays indicated that **5** exhibited the most potent antioxidant effects as measured by DPPH and ABTS assays. In the FRAP experiment, **3** showed the most potent antioxidant effect (3898.88 ± 23.23 mM TE/g). Compounds **2**–**4** had a stronger anti-α-glucosidase effect than acarbose, and compounds **2**, **3**, and **5** showed better anti-AChE activity than chlorogenic acid. In addition, compounds **3**, **4**, and **5** obviously inhibited iNOS and NO production in a concentration-dependent manner. Compounds **3**, **4**, and **5** also significantly inhibited the production of TNF-α and IL-6 in a concentration-dependent manner. This verifies that these compounds possess potent anti-inflammatory activity. Further molecular docking computing results supported that the binding affinity of rutin (**5**) was significantly higher than compounds **1**–**4** and the positive control quercetin. This revealed that **5** has the strongest binding affinity with iNOS, which is consistent with the highest anti-NO activity of **5**.

The above active extracts and active compounds (especially **3**–**5**) can be applied as herbal antioxidants against oxidative damage, and scopoletin (**2**) may be used as a natural anti-α-glucosidase agent. Furthermore, rutin (**5**) and isoquercetin (**3**) also can be used as natural anti-inflammatory and anti-AChE agents.

## Figures and Tables

**Table 1:** Total phenol contents (TPC), total flavonoid contents (TFC) and extraction yields of different solvent extracts from *Polygonatum sibiricum*.

Extracting Solvents	TPC (mg/g) ^a^ (GAE)		TFC (mg/g) ^b^ (QCE)		Yields (%) ^c^	
	PS	PPS	PS	PPS	PS	PPS
Dichloromethane	77.50 ± 7.34 *	52.52 ± 0.47 **	65.15 ± 6.51 ***	26.86 ± 5.81 **	0.35 ± 0.12	1.25 ± 0.03
Ethyl acetate	54.84 ± 4.56 *	60.47 ± 1.91 *	86.02 ± 1.54 ***	98.30 ± 0.47 ***	1.23 ± 0.50	2.25 ± 0.01
Acetone	75.61 ± 7.51 *	53.11 ± 2.26 **	25.21 ± 5.84 ***	20.70 ± 4.56 **	0.54 ± 0.06	3.75 ± 0.15
Ethanol	50.82 ± 7.56 *	26.52 ± 1.62 *	14.51 ± 3.27 *	19.66 ± 4.38 **	5.32 ± 0.58	5.60 ± 0.89
Methanol	46.37 ± 5.44 *	28.92 ± 2.46 **	9.94 ± 1.34 ***	22.02 ± 4.44 **	13.49 ± 1.53	10.45 ± 1.57
Water	36.87 ± 3.86 **	32.55 ± 2.34 **	12.22 ± 3.47 ***	21.13 ± 2.51 ***	5.36 ± 0.97	15.90 ± 2.64

^a^ TPC was displayed in mg of gallic acid equivalents (GAE) per gram of fresh extract. ^b^ TFC was displayed in mg of quercetin equivalents (QCE) per gram of fresh extract. ^c^ Yield was calculated as % yield = (weight of fresh extract/initial weight of dry sample) × 100. Values are expressed as means ± SD (*n* = 3). *** *p* < 0.001, ** *p* < 0.01, and * *p* < 0.05 compared with the control. PS means *Polygonatum sibiricum*. PPS means processed *Polygonatum sibiricum*.

**Table 2:** Antioxidant effects of different solvent extracts from *Polygonatum sibiricum* measured by DPPH, ABTS, superoxide, and FRAP assays.

Extracting Solvents	SC_50_ (μg/mL) ^a^						TE (mM/g) ^c^	
	DPPH		ABTS		Superoxide		FRAP	
	PS	PPS	PS	PPS	PS	PPS	PS	PPS
Dichloromethane	236.14 ± 3.89 ***	>400	240.49 ± 5.68 ***	>400	>400	>400	667.08 ± 18.56 **	322.26 ± 5.46 *
Ethyl acetate	>400	>400	>400	>400	190.23 ± 1.09 ***	>400	651.03 ± 20.56 **	604.38 ± 3.73 *
Acetone	278.31 ± 3.26 ***	>400	229.37 ± 5.59 ***	>400	>400	>400	515.84 ± 24.86 **	362.87 ± 7.48 *
Ethanol	>400	>400	245.48 ± 3.59 ***	>400	>400	>400	431.13 ± 18.70 **	234.39 ± 1.75 **
Methanol	>400	>400	346.48 ± 3.21 ***	>400	>400	>400	414.40 ± 17.74 **	203.03 ± 4.08 *
Water	>400	>400	>400	>400	294.54 ± 7.28 **	>400	296.71 ± 16.46 *	116.01 ± 2.11 *
BHT ^b^	35.54 ± 0.64 **		20.57 ± 0.22 **		N.A. ^d^		3005.93 ± 13.10 ***	

^a^ SC_50_ value was defined as the concentration of the samples causing 50% free radical scavenging and was displayed as mean ± SD (*n* = 3). ^b^ Butylated hydroxytoluene (BHT) was used as a positive control. ^c^ Ferric reducing antioxidant power (FRAP) assay was displayed as millimolar (mM) of Trolox equivalents (TE) per gram of fresh extract. ^d^ N.A. indicates not available (poor solubility). *** *p* < 0.001, ** *p* < 0.01, and * *p* < 0.05 compared with the control. PS means *Polygonatum sibiricum*. PPS means processed *Polygonatum sibiricum*.

**Table 3:** Anti-α-glucosidase and acetylcholinesterase inhibitory effects of different solvent extracts.

Extracting Solvents	α-Glucosidase IC_50_ (μg/mL) ^a^		AChE IC_50_ (μg/mL) ^a^	
	PS	PPS	PS	PPS
Dichloromethane	34.29 ± 6.26 *	>600	60.90 ± 6.18 **	56.27 ± 7.11 ***
Ethyl acetate	22.34 ± 1.66 **	>600	86.91 ± 3.25 **	32.60 ± 5.27 *
Acetone	26.13 ± 2.48 ***	>600	65.54 ± 10.70 **	68.45 ± 4.95 ***
Ethanol	>600	>600	63.41 ± 7.58 **	>400
Methanol	>600	>600	65.59 ± 5.83 **	>400
Water	>600	>600	94.07 ± 5.00	>400
Acarbose ^b^	379.07 ± 4.23 *		—	
Chlorogenic acid ^b^	—		23.27 ± 0.10 *	

^a^ The IC_50_ value was defined as half-maximal inhibitory concentration, and was expressed as mean ± SD (*n* = 3). ^b^ Acarbose and chlorogenic acid were applied as positive controls. *** *p* < 0.001, ** *p* < 0.01, and * *p* < 0.05 compared with the control. PS means *Polygonatum sibiricum*. PPS means processed *Polygonatum sibiricum*.

**Table 4:** Nitric oxide inhibitory effects of various solvent extracts.

Extracting Solvents	Nitric Oxide IC_50_ (μg/mL) ^a^	
	PS	PPS
Dichloromethane	18.84 ± 1.80 **	27.48 ± 6.99 **
Ethyl acetate	45.22 ± 6.80 **	61.08 ± 2.88 **
Acetone	40.68 ± 6.13 **	105.94 ± 8.63 *
Ethanol	81.23 ± 2.26 *	181.80 ± 7.63 *
Methanol	91.14 ± 8.18 *	157.43 ± 9.56 *
Water	176.82 ± 8.64 *	202.85 ± 19.41 *
Quercetin ^b^	7.52 ± 0.25 *	

^a^ The IC_50_ value was defined as half-maximal inhibitory concentration, and was expressed as mean ± SD (*n* = 3). ^b^ Quercetin was applied as positive control. ** *p* < 0.01, and * *p* < 0.05 compared with the control. PS means *Polygonatum sibiricum*. PPS means processed *Polygonatum sibiricum*.

**Table 5:** Quantification of the major bioactive compounds from *Polygonatum sibiricum* in various solvent extracts.

Extracting Solvents	5-HMF (mg/g)	Scopoletin (mg/g)	Rutin (mg/g)	Hyperoside (mg/g)	Isoquercetin (mg/g)	Total Amount (mg/g)
Water (PS)	3.73 ± 0.22	N.D. ^a^	1.80 ± 0.18	N.D. ^a^	1.24 ± 0.10	6.77 ± 0.50
Methanol (PS)	5.73 ± 0.36	1.17 ± 0.08	1.60 ± 0.11	2.14 ± 0.22	2.66 ± 0.13	13.30 ± 0.90
Ethanol (PS)	6.43 ± 0.48	2.85 ± 0.19	1.51 ± 0.10	1.44 ± 0.09	1.26 ± 0.13	13.49 ± 0.99
Acetone (PS)	5.23 ± 0.61	2.33 ± 0.22	1.24 ± 0.09	1.21 ± 0.16	1.02 ± 0.06	11.03 ± 1.14
Ethyl acetate (PS)	3.84 ± 0.22	4.69 ± 0.35	1.32 ± 0.07	2.34 ± 0.28	1.22 ± 0.08	13.41 ± 1.00
Dichloromethane (PS)	5.63 ± 0.48	2.12 ± 0.34	3.21 ± 0.33	1.93 ± 0.09	4.36 ± 0.44	17.25 ± 1.68
Water (PPS)	12.81 ± 2.38	N.D. ^a^	1.30 ± 0.07	1.24 ± 0.09	1.84 ± 0.12	17.19 ± 2.66
Methanol (PPS)	20.83 ± 1.84	N.D. ^a^	1.82 ± 0.11	1.24 ± 0.13	1.74 ± 0.18	25.63 ± 2.26
Ethanol (PPS)	22.43 ± 2.12	N.D. ^a^	N.D. ^a^	3.64 ± 0.32	N.D. ^a^	26.07 ± 2.44
Acetone (PPS)	24.63 ± 1.92	N.D. ^a^	N.D. ^a^	2.26 ± 0.13	N.D. ^a^	26.89 ± 2.05
Ethyl acetate (PPS)	21.86 ± 2.13	N.D. ^a^	N.D. ^a^	3.44 ± 0.31	N.D. ^a^	25.03 ± 2.44
Dichloromethane (PPS)	23.86 ± 1.88	N.D. ^a^	N.D. ^a^	3.63 ± 0.43	N.D. ^a^	27.49 ± 2.31

Results are displayed as milligrams of each component in gram of extract. ^a^ N.D. is characterized as not detected (less than LOD); PS means *Polygonatum sibiricum*, and PPS means processed *Polygonatum sibiricum*.

**Table 6:** Antioxidant effects of isolated components from *Polygonatum sibiricum* measured by DPPH, ABTS, superoxide, and FRAP assays.

Compounds	SC_50_ (μM) ^a^			(mM/g) (TE) ^c^
	DPPH	ABTS	Superoxide	FRAP
5-HMF (**1**)	>400	>400	>400	26.70 ± 0.97 *
Scopoletin (**2**)	>400	91.27 ± 3.36 *	>400	2892.97 ± 19.18 ***
Isoquercetin (**3**)	12.64 ± 3.21 *	22.73 ± 1.17 *	179.62 ± 4.43 **	3898.88 ± 23.23 ***
Hyperoside (**4**)	12.46 ± 4.02 *	29.26 ± 0.51 *	172.50 ± 3.80 **	3246.93 ± 31.92 ***
Rutin (**5**)	5.60 ± 0.34 ***	15.43 ± 0.25 **	174.82 ± 3.02 **	2221.33 ± 5.02 ***
BHT ^b^	192.28 ± 8.94 *	100.35 ± 7.26 *	N.A. ^d^	2896.93 ± 21.19 ***

^a^ SC_50_ value was defined as the concentration of the samples causing 50% free radical scavenging and was displayed as mean ± SD (*n* = 3). ^b^ Butylated hydroxytoluene (BHT) was used as a positive control. ^c^ Ferric reducing antioxidant power (FRAP) assay was displayed as millimolar (mM) of Trolox equivalents (TE) per gram of extract. ^d^ N.A. indicates not available (poor solubility). *** *p* < 0.001, ** *p* < 0.01, and * *p* < 0.05 compared with the control.

**Table 7:** Anti-α-glucosidase and anti-acetylcholinesterase effects of isolated compounds.

Compounds	α-Glucosidase	AChE
	IC_50_ (μM) ^a^	
5-HMF (**1**)	>600	81.46 ± 11.05 **
Scopoletin (**2**)	23.63 ± 7.22 ***	32.35 ± 2.05 **
Isoquercetin (**3**)	159.73 ± 3.12 ***	23.13 ± 3.15 ***
Hyperoside (**4**)	208.14 ± 5.70 ***	121.10 ± 10.70 **
Rutin (**5**)	331.15 ± 3.81 **	33.09 ± 5.43 **
Acarbose ^b^	550.15 ± 7.65 *	—
Chlorogenic acid ^b^	—	68.23 ± 2.90 *

^a^ The IC_50_ value was defined as half-maximal inhibitory concentration, and was expressed as mean ± SD (*n* = 3). ^b^ Acarbose and chlorogenic acid were used as positive controls for anti-α-glucosidase and anti-AChE assays, respectively. *** *p* < 0.001, ** *p* < 0.01, and * *p* < 0.05 compared with the control.

**Table 8:** Nitric oxide inhibitory assay of isolated compounds.

Compounds	Nitric Oxide
	IC_50_ (μM) ^a^
5-HMF (**1**)	34.90 ± 8.80 *
Scopoletin (**2**)	36.26 ± 4.65 *
Isoquercetin (**3**)	17.03 ± 1.28 **
Hyperoside (**4**)	18.87 ± 1.68 *
Rutin (**5**)	9.89 ± 1.36 **
Quercetin ^b^	18.26 ± 0.54 *

^a^ The IC_50_ value was defined as half-maximal inhibitory concentration, and was expressed as mean ± SD (*n* = 3). ^b^ Quercetin was applied as a positive control. ** *p* < 0.01, and * *p* < 0.05 compared with the control.

**Table 9:** Binding energies of active compounds and chlorogenic acid with AChE calculated in silico.

Compounds	Affinity (kcal/mol)
5-HMF (**1**)	−4.8
Scopoletin (**2**)	−7.0
Isoquercetin (**3**)	−7.5
Hyperoside (**4**)	−3.2
Rutin (**5**)	−6.8
Chlorogenic acid ^a^	−5.3

^a^ Chlorogenic acid was used as a positive control.

**Table 10:** Binding energies of active compounds and quercetin with iNOS calculated in silico.

Compounds	Affinity (kcal/mol)
5-HMF (**1**)	−5.8
Scopoletin (**2**)	−5.5
Isoquercetin (**3**)	−7.3
Hyperoside (**4**)	−6.8
Rutin (**5**)	−9.5
Quercetin ^a^	−7.9

^a^ Quercetin was used as a positive control.

## Data Availability

The data presented in this study are available in the main text and the Supplementary Materials of this article.

## Supplementary Materials

The following are available online at https://www.mdpi.com/article/10.3390/antiox11071383/s1, Table S1: Retention time, LODs, LOQs, and regression analysis for five components of *Polygonum sibiricum* in reversed-phase HPLC. Figure S1: The ^1^H-NMR spectrum of 5-HMF (**1**). Figure S2: The ^1^H-NMR spectrum of scopoletin (**2**). Figure S3: The ^1^H-NMR spectrum of isoquercetin (**3**). Figure S4: ^1^H-NMR spectrum of hyperoside (**4**). Figure S5: ^1^H-NMR spectrum of rutin (**5**). Figure S6: Reversed-phase HPLC chromatogram of isolated pure compounds. Figure S7: Reversed-phase HPLC chromatogram of water extract in PS. Figure S8: Reversed-phase HPLC chromatogram of methanol extract in PS. Figure S9: Reversed-phase HPLC chromatogram of ethanol extract in PS. Figure S10: Reversed-phase HPLC chromatogram of acetone extract in PS. Figure S11: Reversed-phase HPLC chromatogram of ethyl acetate extract in PS. Figure S12: Reversed-phase HPLC chromatogram of dichloromethane extract in PS. Figure S13: Reversed-phase HPLC chromatogram of water extract in PPS. Figure S14: Reversed-phase HPLC chromatogram of methanol extract in PPS. Figure S15: Reversed-phase HPLC chromatogram of ethanol extract in PPS. Figure S16: Reversed-phase HPLC chromatogram of acetone extract in PPS. Figure S17: Reversed-phase HPLC chromatogram of ethyl acetate extract in PPS. Figure S18: Reversed-phase HPLC chromatogram of dichloromethane extract in PPS. Figure S19: Interactions of rutin (**5**) with active sites of *E. electricus* AChE.

## References

[1] Gong P.-Y., Guo Y.-J., Tian Y.-S., Gu L.-F., Qi J., Yu B.-Y. (2021). Reverse tracing anti-thrombotic active ingredients from dried *Rehmannia Radix* based on multidimensional spectrum-effect relationship analysis of steaming and drying for nine cycles. J. Ethnopharmacol..

[2] Zhang H., Hao F., Yao Z., Zhu J., Jing X., Wang X. (2022). Efficient extraction of flavonoids from *Polygonatum sibiricum* using a deep eutectic solvent as a green extraction solvent. Microchem. J..

[3] Chen H., Li Y.J., Li X.F., Sun Y.J., Li H.W., Su F.Y., Feng W.S. (2018). Homoisoflavanones with estrogenic activity from the rhizomes of *Polygonatum sibiricum*. J. Asian Nat. Prod. Res..

[4] Sun L.R., Li X., Wang S.X. (2005). Two new alkaloids from the rhizome of *Polygonatum sibiricum*. J. Asian Nat. Prod. Res..

[5] Son K.H., Do J.C., Kang S.S. (1990). Steroidal saponins from the rhizomes of *Polygonatum sibiricum*. J. Nat. Prod..

[6] Hu C.Y., Xu D.P., Wu Y.M., Ou S.Y. (2010). Triterpenoid saponins from the rhizome of *Polygonatum sibiricum*. J. Asian Nat. Prod. Res..

[7] Zheng S. (2020). Protective effect of *Polygonatum sibiricum* Polysaccharide on D-galactose-induced aging rats model. Sci. Rep..

[8] QIN Z. (2019). Effect of rhizoma *polygonati* on functional activity of endothelial progenitor cells to delay senescense via decrease of ros. Chin. Pharmacol. Bull..

[9] Debnath T., Park S.R., Jo J.E., Lim B.O. (2013). Antioxidant and anti-inflammatory activity of *Polygonatum sibiricum* rhizome extracts. Asian Pacific J. Trop. Dis..

[10] Zhao H., Wang Q.-L., Hou S.-B., Chen G. (2019). Chemical constituents from the rhizomes of *Polygonatum sibiricum* Red. and anti-inflammatory activity in RAW264. 7 macrophage cells. Nat. Prod. Res..

[11] Liu J., Li T., Chen H., Yu Q., Yan C. (2021). Structural characterization and osteogenic activity in vitro of novel polysaccharides from the rhizome of *Polygonatum sibiricum*. Food Funct..

[12] Du L., Nong M.-N., Zhao J.-M., Peng X.-M., Zong S.-H., Zeng G.-F. (2016). *Polygonatum sibiricum* polysaccharide inhibits osteoporosis by promoting osteoblast formation and blocking osteoclastogenesis through Wnt/β-catenin signalling pathway. Sci. Rep..

[13] He Y., Huang L., Jiang P., Xu G., Sun T. (2022). Immunological regulation of the active fraction from *Polygonatum sibiricum* F. Delaroche based on improvement of intestinal microflora and activation of RAW264. 7 cells. J. Ethnopharmacol..

[14] Liu N., Dong Z., Zhu X., Xu H., Zhao Z. (2018). Characterization and protective effect of *Polygonatum sibiricum* polysaccharide against cyclophosphamide-induced immunosuppression in Balb/c mice. Int. J. Biol. Macromol..

[15] Huang S., Yuan H., Li W., Liu X., Zhang X., Xiang D., Luo S. (2021). *Polygonatum sibiricum* polysaccharides protect against MPP-induced neurotoxicity via the Akt/mTOR and Nrf2 pathways. Oxid. Med. Cell. Longev..

[16] Yu L.-Z., Zhang X.-P., Wang Y.-X. (2019). *Polygonatum sibiricum* extract exerts inhibitory effect on diabetes in a rat model. Trop. J. Pharm. Res..

[17] Xie Y., Jiang Z., Yang R., Ye Y., Pei L., Xiong S., Wang S., Wang L., Liu S. (2021). Polysaccharide-rich extract from *Polygonatum sibiricum* protects hematopoiesis in bone marrow suppressed by triple negative breast cancer. Biomed. Pharmacother..

[18] Jo K., Suh H.J., Choi H.S. (2018). *Polygonatum sibiricum* rhizome promotes sleep by regulating non-rapid eye movement and GABAergic/serotonergic receptors in rodent models. Biomed. Pharmacother..

[19] Li M., Jiang H., Hao Y., Du K., Du H., Ma C., Tu H., He Y. (2021). A systematic review on botany, processing, application, phytochemistry and pharmacological action of *Radix Rehmnniae*. J. Ethnopharmacol..

[20] Shapla U.M., Solayman M., Alam N., Khalil M., Gan S.H. (2018). 5-Hydroxymethylfurfural (HMF) levels in honey and other food products: Effects on bees and human health. Chem. Cent. J..

[21] Droadowski L.A., Thomson A.B. (2006). Intestinal sugar transport. World J. Gastroenterol..

[22] Proença C., Freitas M., Ribeiro D., Oliveira E.F., Sousa J.L., Tomé S.M., Ramos M.J., Silva A.M., Fernandes P.A., Fernandes E. (2017). α-Glucosidase inhibition by flavonoids: An in vitro and in silico structure activity relationship study. J. Enzym. Inhib. Med. Chem..

[23] Reddy V.P., Zhu X., Perry G., Smith M.A. (2009). Oxidative stress in diabetes and Alzheimer’s disease. J. Alzheimers Dis..

[24] Kwon S.-H., Lee H.-K., Kim J.-A., Hong S.-I., Kim H.-C., Jo T.-H., Park Y.-I., Lee C.-K., Kim Y.-B., Lee S.-Y. (2010). Neuroprotective effects of chlorogenic acid on scopolamine-induced amnesia via anti-acetylcholinesterase and anti-oxidative activities in mice. Eur. J. Pharmacol..

[25] Van’T Hof R.J., Ralston S.H. (2001). Nitric oxide and bone. Immunology.

[26] Hämäläinen M., Nieminen R., Vuorela P., Heinonen M., Moilanen E. (2007). Anti-inflammatory effects of flavonoids: Genistein, kaempferol, quercetin, and daidzein inhibit STAT-1 and NF-κB activations, whereas flavone, isorhamnetin, naringenin, and pelargonidin inhibit only NF-κB activation along with their inhibitory effect on iNOS expression and NO production in activated macrophages. Mediat. Inflamm..

[27] Liang Y.-C., Huang Y.-T., Tsai S.-H., Lin-Shiau S.-Y., Chen C.-F., Lin J.-K. (1999). Suppression of inducible cyclooxygenase and inducible nitric oxide synthase by apigenin and related flavonoids in mouse macrophages. Carcinogenesis.

[28] Chu Y.C., Yang C.S., Cheng M.J., Fu S.L., Chen J.J. (2022). Comparison of various solvent extracts and major bioactive components from unsalt-fried and salt-fried rhizomes of *Anemarrhena asphodeloides* for antioxidant, anti-α-glucosidase, and an-ti-acetylcholinesterase activities. Antioxidants.

[29] Lin Y.T., Lin H.R., Yang C.S., Liaw C.C., Sung P.J., Kuo Y.H., Cheng M.J., Chen J.J. (2022). Antioxidant and Anti-α-glucosidase activities of various solvent extracts and major bioactive components from the fruits of *Crataegus pinnatifida*. Antioxidants.

[30] Noreen H., Semmar N., Farman M., McCullagh J.S. (2017). Measurement of total phenolic content and antioxidant activity of aerial parts of medicinal plant *Coronopus didymus*. Asian Pac. J. Trop. Med..

[31] Do Q.D., Angkawijaya A.E., Tran-Nguyen P.L., Huynh L.H., Soetaredjo F.E., Ismadji S., Ju Y.-H. (2014). Effect of extraction solvent on total phenol content, total flavonoid content, and antioxidant activity of *Limnophila aromatica*. J. Food Drug Anal..

[32] Sharma S.K., Singh A.P. (2012). In vitro antioxidant and free radical scavenging activity of *Nardostachys jatamansi* DC. J. Acupunct. Meridian Stud..

[33] Re R., Pellegrini N., Proteggente A., Pannala A., Yang M., Rice-Evans C. (1999). Antioxidant activity applying an improved abts radical cation decolorization assay. Free Radic. Biol. Med..

[34] Benzie I.F., Strain J.J. (1996). The ferric reducing ability of plasma (FRAP) as a measure of “antioxidant power”: The FRAP assay. Anal. Biochem..

[35] Kim Y.-M., Wang M.-H., Rhee H.-I. (2004). A novel α-glucosidase inhibitor from pine bark. Carbohydr. Res..

[36] Tran T.-D., Nguyen T.-C.-V., Nguyen N.-S., Nguyen D.-M., Nguyen T.-T.-H., Le M.-T., Thai K.-M. (2016). Synthesis of novel chalcones as acetylcholinesterase inhibitors. Appl. Sci..

[37] Lee C.J., Lee S.S., Chen S.C., Ho F.M., Lin W.W. (2005). Oregonin inhibits lipopolysaccharide-induced iNOS gene transcription and upregulates HO-1 expression in macrophages and microglia. Br. J. Pharmacol..

[38] Trott O., Olson A.J. (2010). AutoDock Vina: Improving the speed and accuracy of docking with a new scoring function, efficient optimization, and multithreading. J. Comput. Chem..

[39] BIOVIA (2021). Dassault Systèmes. Discovery Studio Client 2021, v.21.1.0.

[40] Oboh G., Agunloye O.M., Akinyemi A.J., Ademiluyi A.O., Adefegha S.A. (2013). Comparative study on the inhibitory effect of caffeic and chlorogenic acids on key enzymes linked to Alzheimer’s disease and some pro-oxidant induced oxidative stress in rats’ brain-in vitro. Neurochem. Res..

[41] Bourne Y., Grassi J., Bougis P.E., Marchot P. (1999). Conformational flexibility of the acetylcholinesterase tetramer suggested by X-ray crystallography. J. Biol. Chem..

[42] Francis S.M., Mittal A., Sharma M., Bharatam P.V. (2008). Design of Benzene-1,2-diamines as selective inducible nitric oxide synthase inhibitors: A combined de novo design and docking analysis. J. Mol. Model..

[43] Zhang Q.-W., Lin L.-G., Ye W.-C. (2018). Techniques for extraction and isolation of natural products: A comprehensive review. Chin. Med..

[44] Mbeunkui F., Grace M.H., Lategan C., Smith P.J., Raskin I., Lila M.A. (2011). Isolation and identification of antiplasmodial N-alkylamides from *Spilanthes acmella* flowers using centrifugal partition chromatography and ESI-ITTOF-MS. J. Chromatogr. B.

[45] Mareček V., Mikyška A., Hampel D., Čejka P., Neuwirthová J., Malachová A., Cerkal R. (2017). ABTS and DPPH methods as a tool for studying antioxidant capacity of spring barley and malt. J. Cereal Sci..

[46] Raghavendra R., Neelagund S., Kuluvar G., Bhanuprakash V., Revanaiah Y. (2010). Protective effect of partially purified 35 kDa protein from silk worm (*Bombyx mori*) fecal matter against carbon tetrachloride induced hepatotoxicity and in vitro anti-viral properties. Pharm. Biol..

[47] Guo C., Yang J., Wei J., Li Y., Xu J., Jiang Y. (2003). Antioxidant activities of peel, pulp and seed fractions of common fruits as determined by FRAP assay. Nutr. Res..

[48] Zaheer J., Najam-Us-Saqib Q., Qamar M., Akram M. (2019). In vitro (anti-alpha-glucosidase) activity and in vivo anti-diabetic activity of *Androsace foliosa* (common rock jasmine) in alloxan-induced diabetic BALB/c mice. Eur. J. Inflamm..

[49] Gao L., Li X., Meng S., Ma T., Wan L., Xu S. (2020). Chlorogenic acid alleviates Aβ25-35-induced autophagy and cognitive impairment via the mTOR/TFEB signaling pathway. Drug Des. Dev. Ther..

[50] Yang Q., Kang Z., Zhang J., Qu F., Song B. (2021). Neuroprotective effects of isoquercetin: An in vitro and in vivo study. Cell J..

