# Supplementary material for: Main Bioactive Components and Their Biological Activities from Natural and Processed Rhizomes of Polygonum sibiricum
Source: Antioxidants (Basel). 2022 Jul 17;11(7):1383. doi: 10.3390/antiox11071383 (PMC9311596; doi:10.3390/antiox11071383)
Supplement: Supplementary file 1 [file antioxidants-11-01383-s001.zip › antioxidants-1812867-supplementary.pdf]

Supplementary Data

Main Bioactive Components and Their Biological Activities from Natural and Processed Rhizomes of *Polygonum sibiricum*

Shih-Chi Chen[1], Chang-Syun Yang [1] and Jih-Jung Chen [1,2,*]

[1] Department of Pharmacy, School of Pharmaceutical Sciences, National Yang Ming Chiao Tung University, Taipei 112304, Taiwan; asd19981214.ps10@nycu.edu.tw (S.-C.C.); tim0619@nycu.edu.tw (C.-S.Y.)

[2] Department of Medical Research, China Medical University Hospital, China Medical University, Taichung 404332, Taiwan

[*] Correspondence: jjungchen@nycu.edu.tw; Tel.: +886-2-2826-7195; Fax: +886-2-2823-2940

Contents

Table S1. Retention time, LODs, LOQs, and regression analysis for five components of *Polygonum sibiricum* in reversed phase HPLC.......... S4

Figure S1. The ^{1}H-NMR spectrum (400 MHz, Methanol-d_4) of 5-HMF (**1**) S5

Figure S2. The ^{1}H-NMR spectrum (600 MHz, DMSO-d_6) of scopoletin (**2**) S5

Figure S3. The ^{1}H-NMR spectrum (600 MHz, Methanol-d_4) of isoquercetin (**3**) S6

Figure S4. The ^{1}H-NMR spectrum (600 MHz, Methanol-d_4) of hyperoside (**4**) S6

Figure S5. The ^{1}H-NMR spectrum (600 MHz, Methanol-d_4) of rutin (**5**) S7

Figure S6. Reversed-phase HPLC chromatogram of isolated pure compounds.......... S8

Figure S7. Reversed-phase HPLC chromatogram of water extract in *Polygonum sibiricum* (PS).......... S8

Figure S8. Reversed-phase HPLC chromatogram of methanol extract in PS.......... S9

Figure S9. Reversed-phase HPLC chromatogram of ethanol extract in PS.......... S9

Figure S10. Reversed-phase HPLC chromatogram of acetone extract in PS.......... S9

Figure S11. Reversed-phase HPLC chromatogram of ethyl acetate extract in PS.......... S10

Figure S12. Reversed-phase HPLC chromatogram of dichloromethane extract in PS.......... S10

Figure S13. Reversed-phase HPLC chromatogram of water extract in processed *Polygonum sibiricum* (PPS).......... S10

Figure S14. Reversed-phase HPLC chromatogram of methanol extract in PPS.......... S11

Figure S15. Reversed-phase HPLC chromatogram of ethanol extract in PPS.......... S11

Figure S16. Reversed-phase HPLC chromatogram of acetone extract in PPS.......... S11

Figure S17. Reversed-phase HPLC chromatogram of ethyl acetate extract in PPS.......... S12

Figure S18. Reversed-phase HPLC chromatogram of dichloromethane extract in PPS...........S12

Figure S19. Interactions of rutin (**5**) with active sites of *E. electricus* AChE............................... S13

Table S1. Retention time, LODs, LOQs, and regression analysis for five components of *Polygonum sibiricum* in reversed phase HPLC.

Compounds	T_m (min) [a]	Regression equation	Correlation coefficient	LOD (μg/mL) [a]	LOQ (μg/mL) [a]
5-HMF (**1**)	9.50	y = 526.24x-693.24	0.9994	3.63	11.02
Scopoletin (**2**)	50.10	y = 268.12x-57.62	0.9999	6.64	20.14
Isoquercetin (**3**)	68.00	y = 140.12x+48.98	0.9998	12.71	38.53
Hyperoside (**4**)	63.00	y = 132.53x+8.73	0.9996	11.95	36.21
Rutin (**5**)	60.00	y = 456.35x+4.53	0.9992	4.62	14.02

[a] Tm: Retention time; LOD: Limit of detection; LOQ: Limit of quantification; LOD was counted as = 3.3 × the standard deviation of the intercept / the slope of the standard curve; LOQ was counted as = 10 × the standard deviation of the intercept / the slope of the standard curve. (LOD and LOQ was calculated with reference to the International Conference on Harmonization's (ICH) content.)

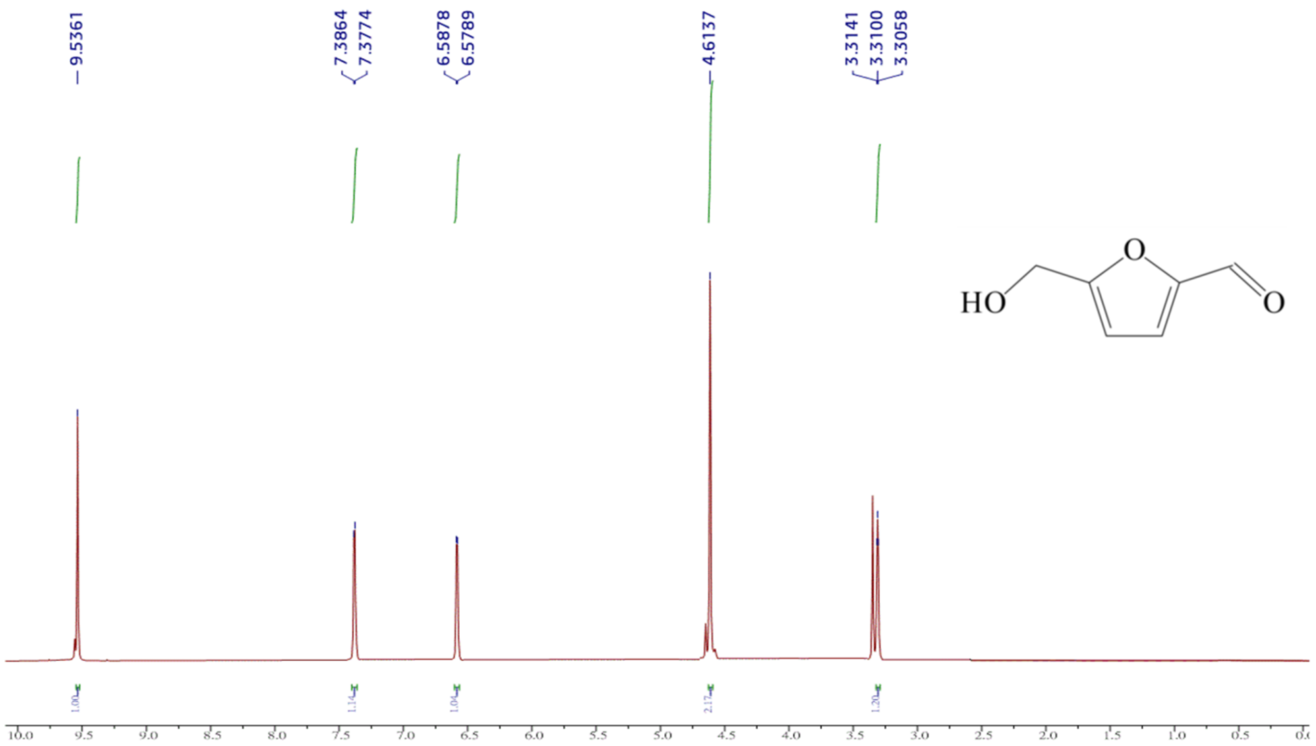

Figure S1. The ^{1}H-NMR spectrum (400 MHz, Methanol-d_4) of 5-HMF (**1**).

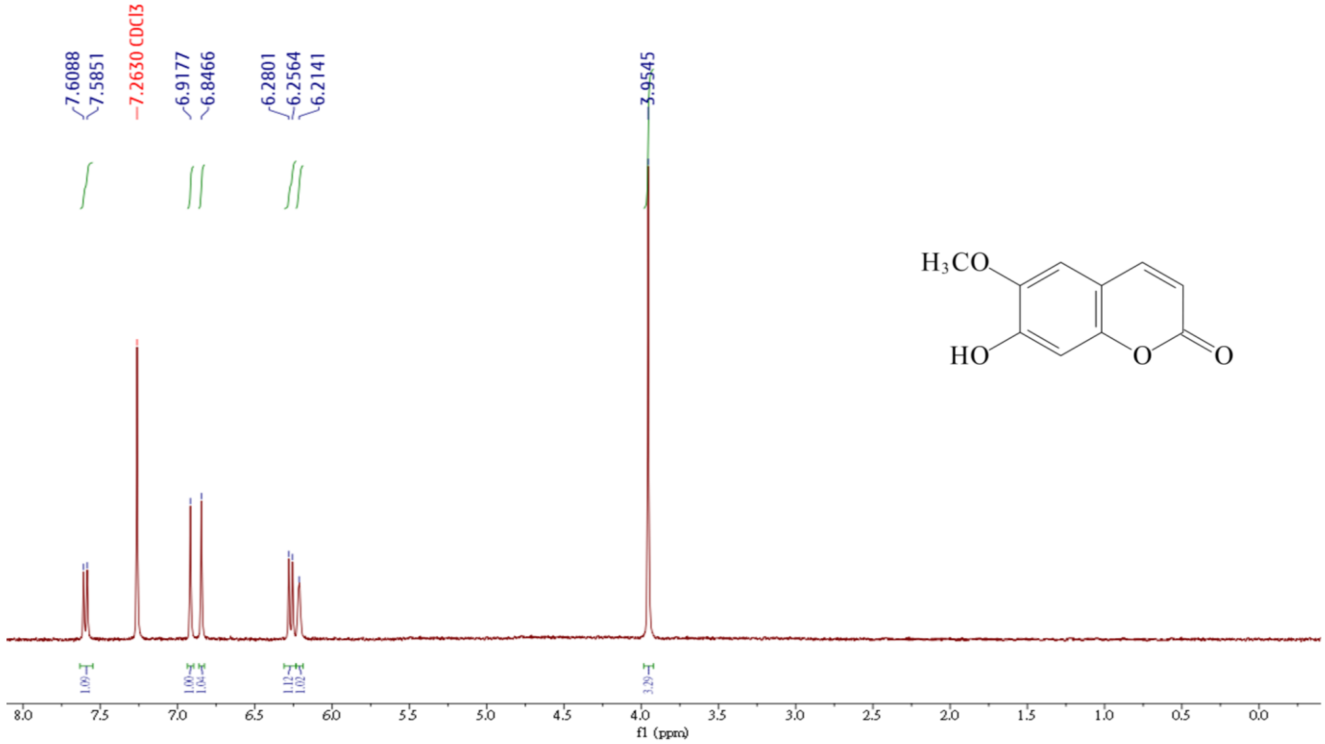

Figure S2. The ^{1}H-NMR spectrum (600 MHz, DMSO-d_6) of scopoletin (**2**).

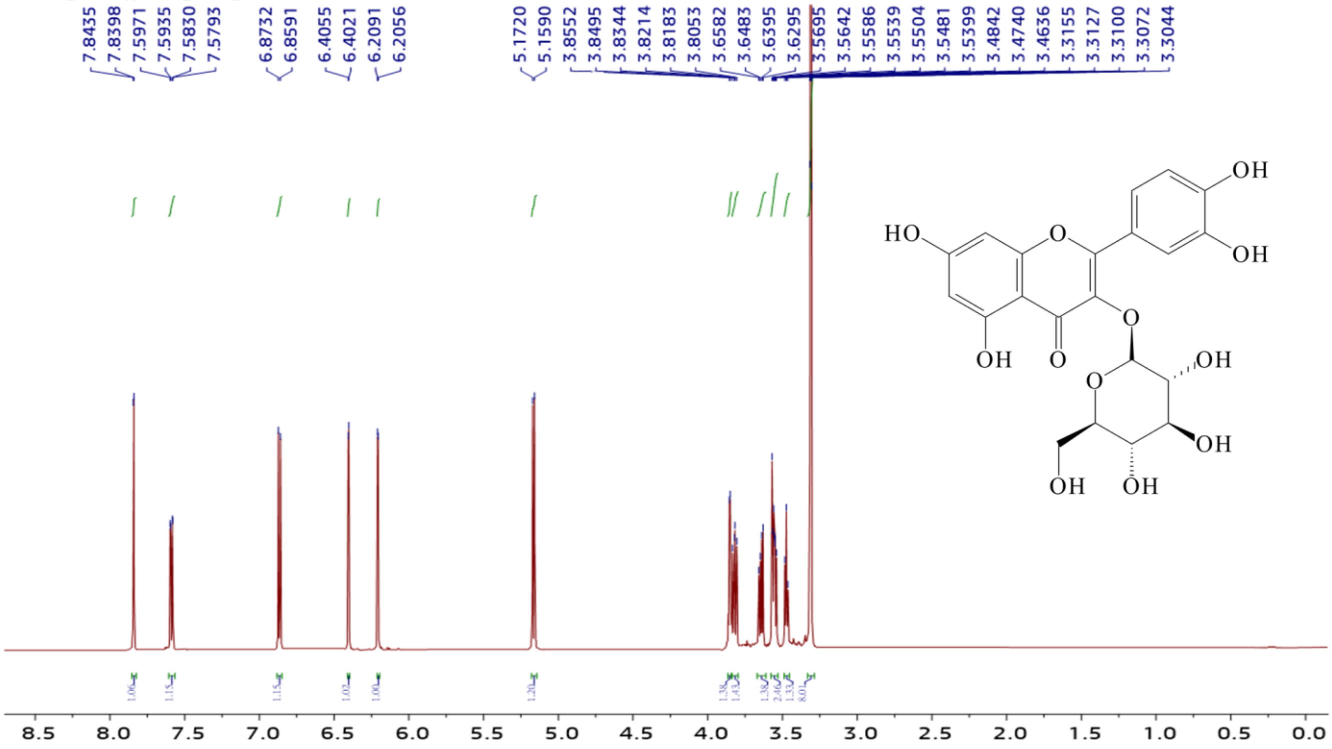

Figure S3. The ^{1}H-NMR spectrum (600 MHz, Methanol-d_4) of isoquercetin (**3**).

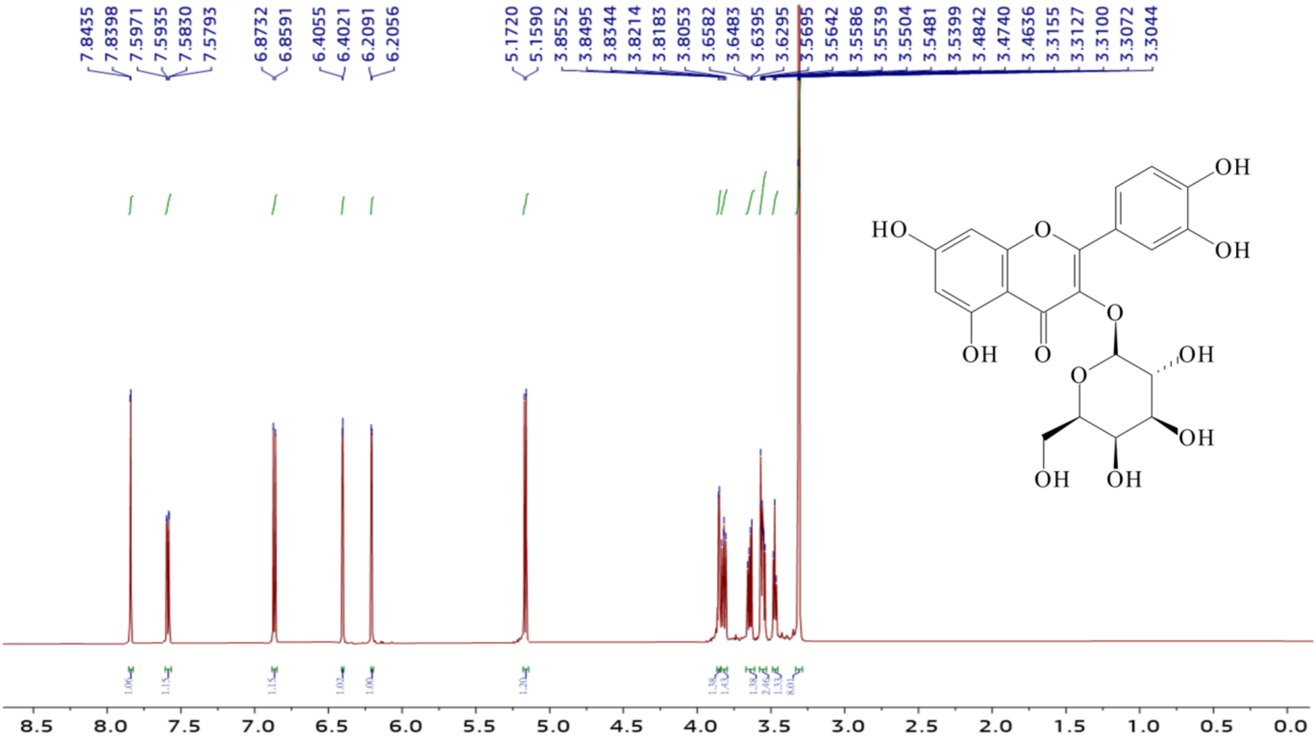

Figure S4. The ^{1}H-NMR spectrum (600 MHz, Methanol-d_4) of hyperoside (**4**)

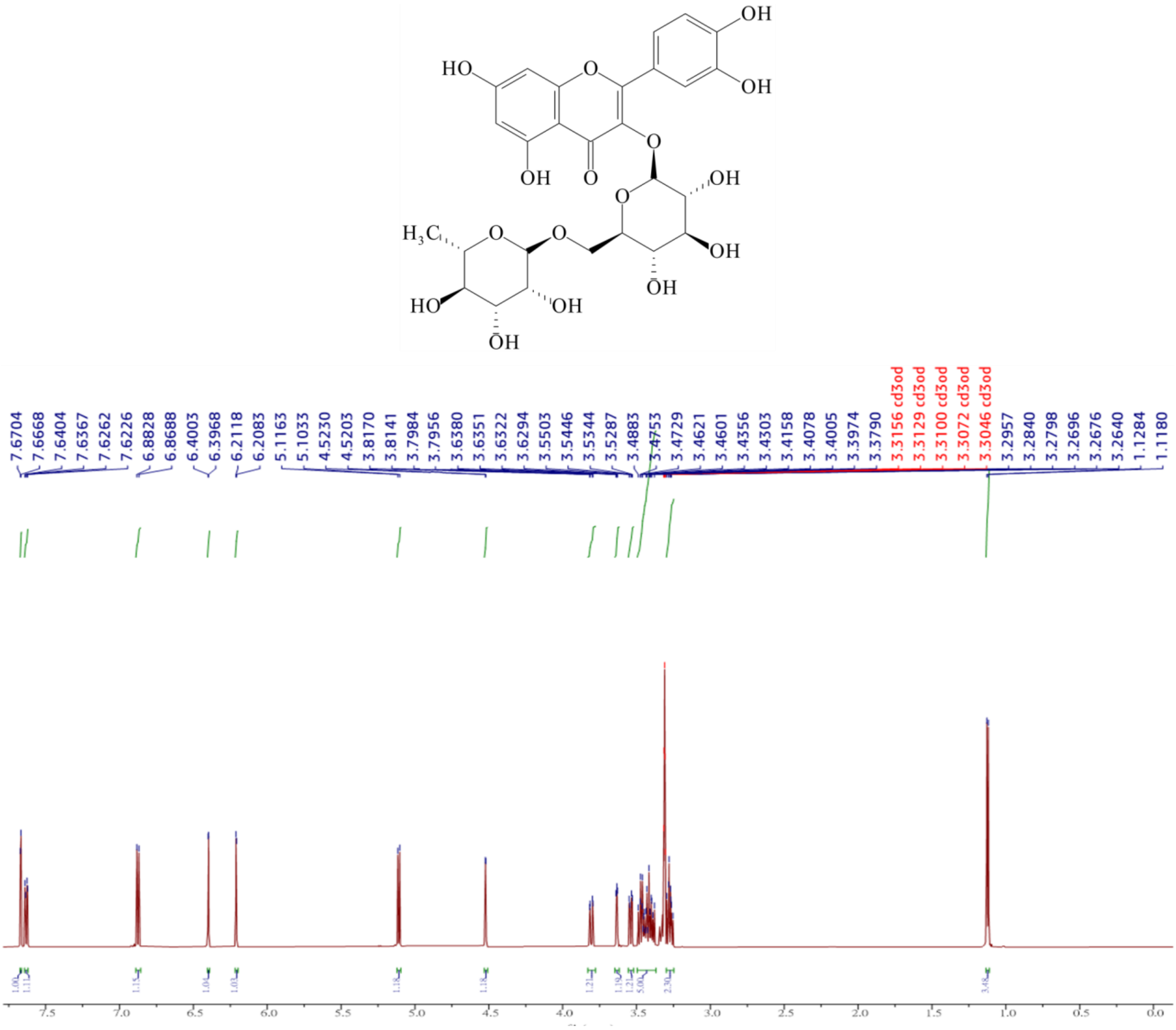

Figure S5. The ^{1}H-NMR spectrum (600 MHz, Methanol-d_4) of rutin (**5**).

(A) 5-HMF (500 μL), 280 nm

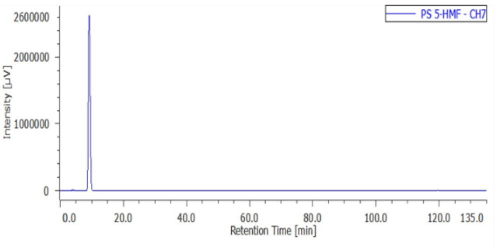

(B) Scopoletin (500 μL), 280 nm

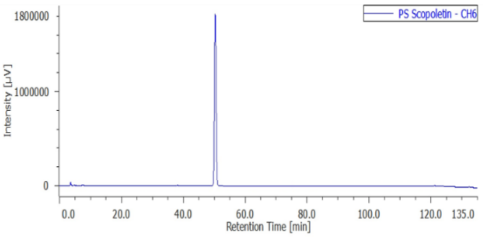

(C) Rutin (500 μL), 280 nm

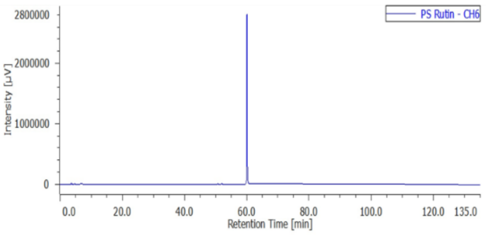

(D) Hyperoside (500 μL), 280 nm

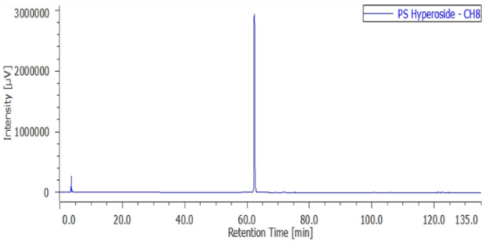

(E) Isoquercetin (500 μL), 280 nm

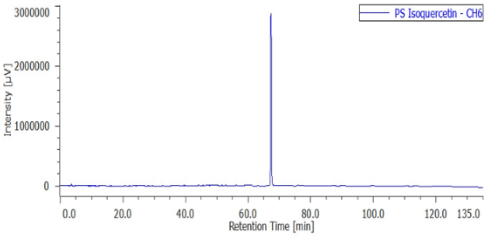

Figure S6. Reversed-phase HPLC chromatogram of isolated pure compounds (A to E).

H_2O (500 μL), 280 nm

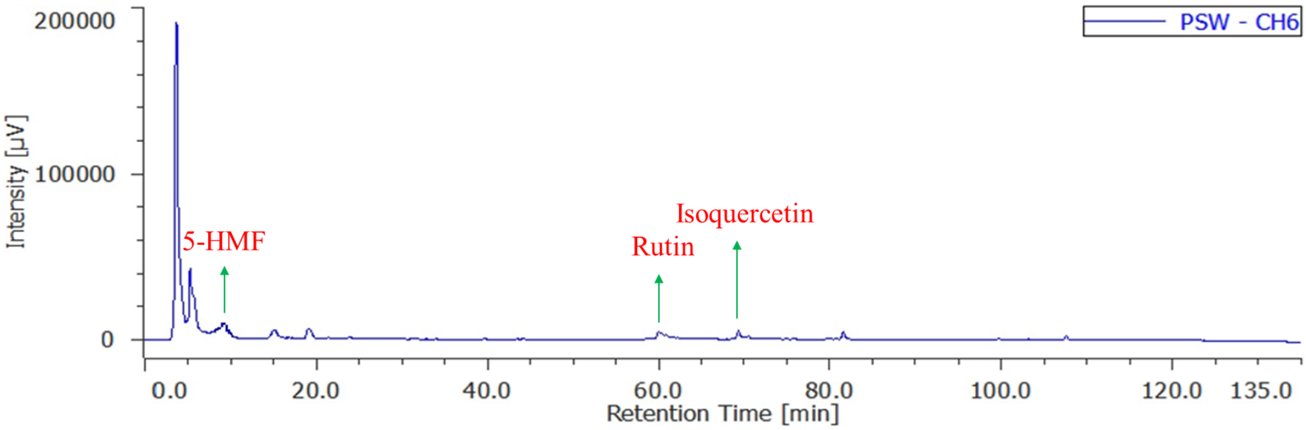

Figure S7. Reversed-phase HPLC chromatogram of water extract in PS.

Methanol (500 µL), 280 nm

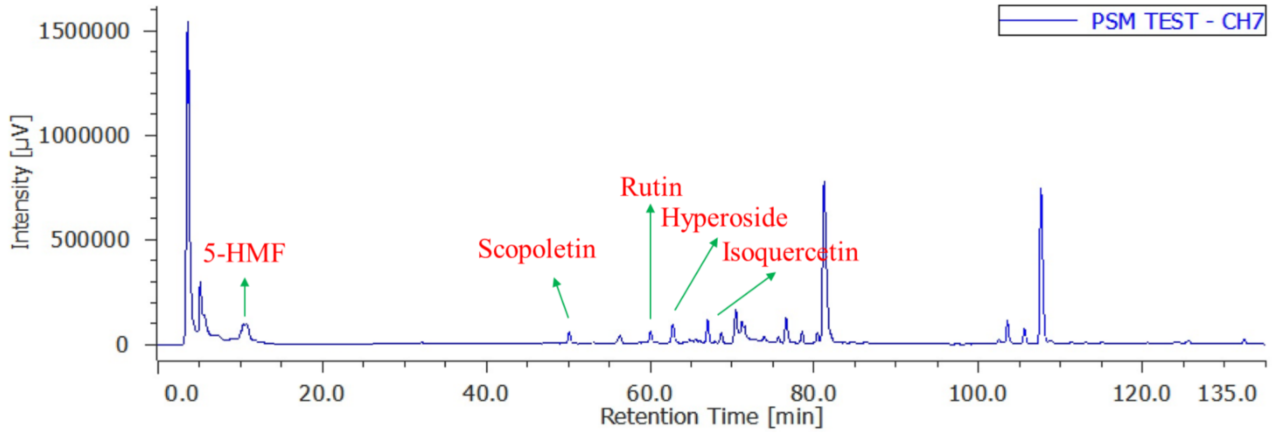

Figure S8. Reversed-phase HPLC chromatogram of methanol extract in PS.

Ethanol (500 µL), 280 nm

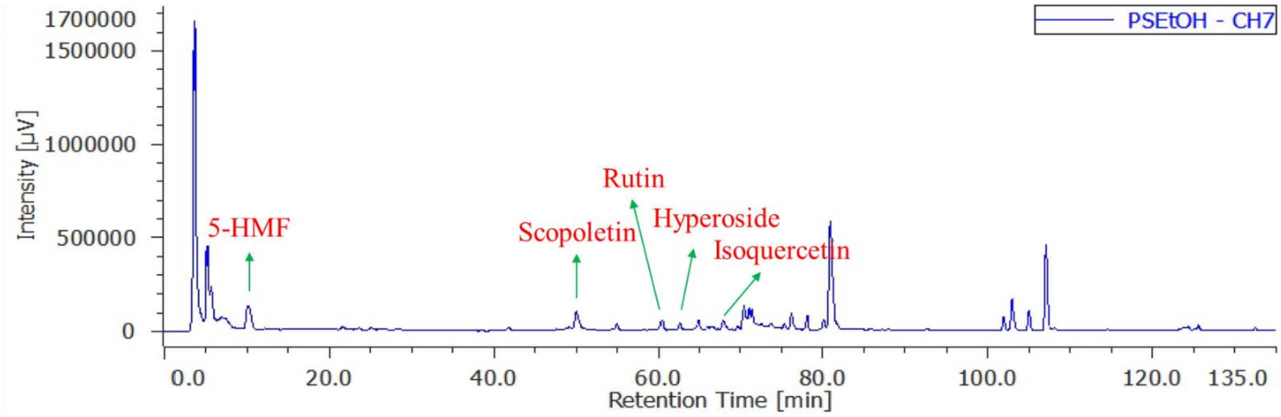

Figure S9. Reversed-phase HPLC chromatogram of ethanol extract in PS.

Acetone (500 µL), 280 nm

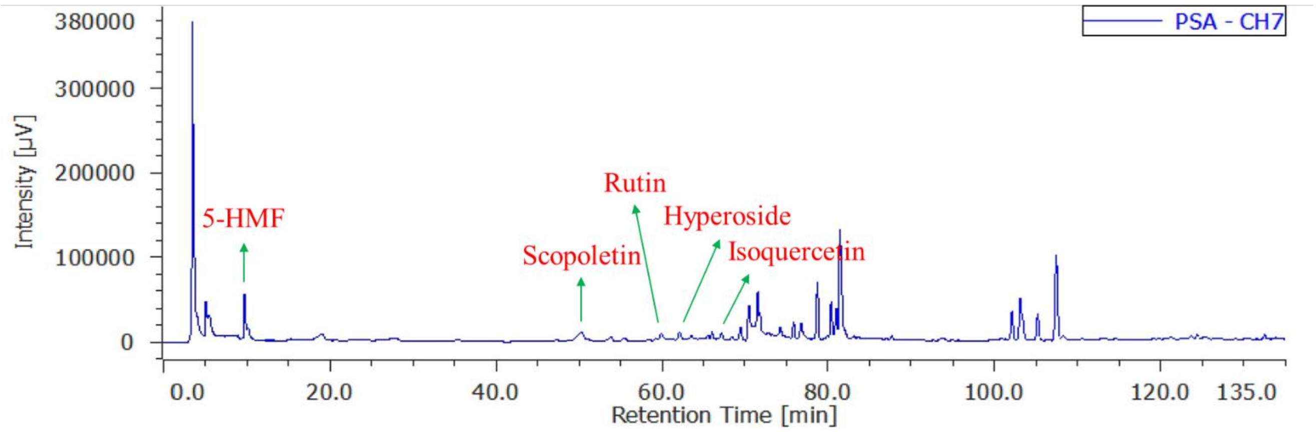

Figure S10. Reversed-phase HPLC chromatogram of acetone extract in PS.

Ethyl acetate (500 µL), 280 nm

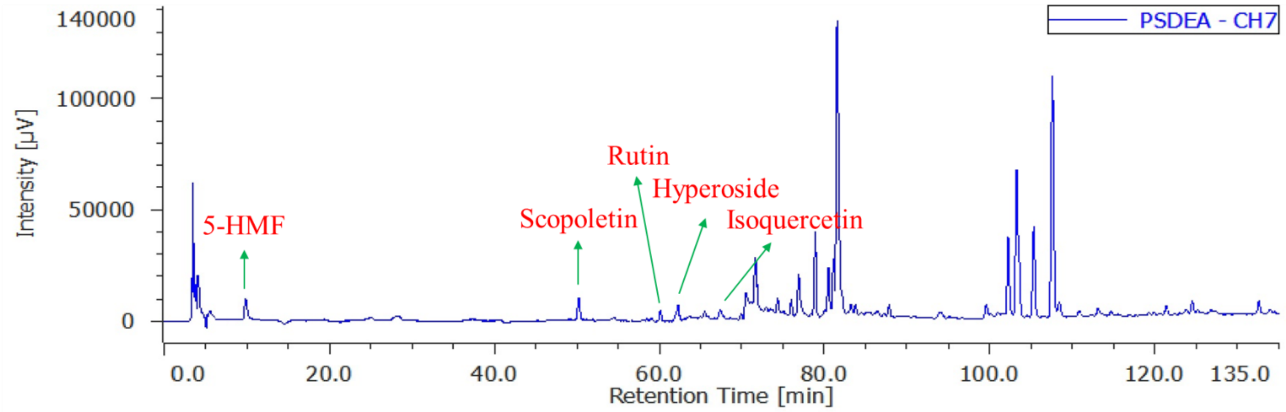

Figure S11. Reversed-phase HPLC chromatogram of ethyl acetate extract in PS.

Dichloromethane (500 µL), 280 nm

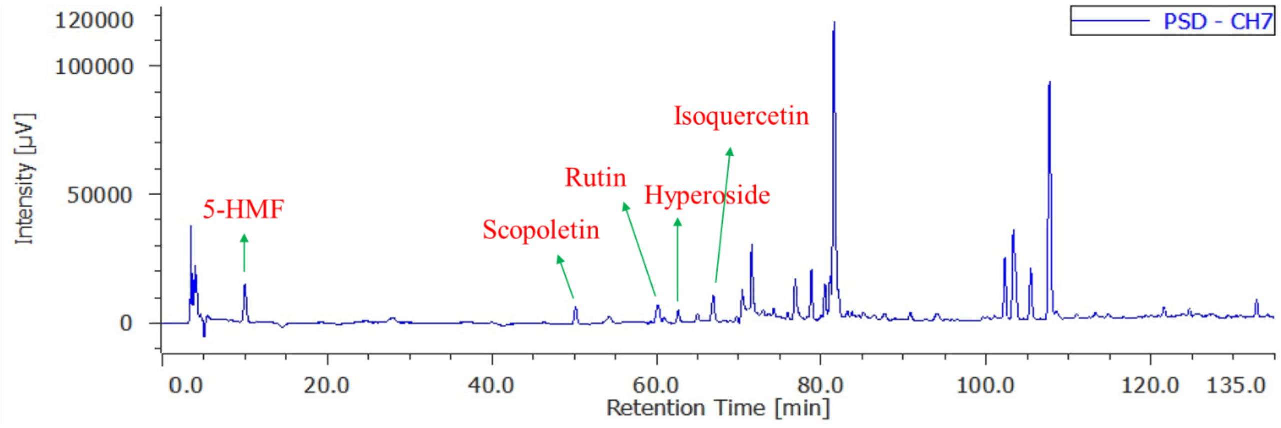

Figure S12. Reversed-phase HPLC chromatogram of dichoromethane extract in PS.

Water (500 µL), 280 nm

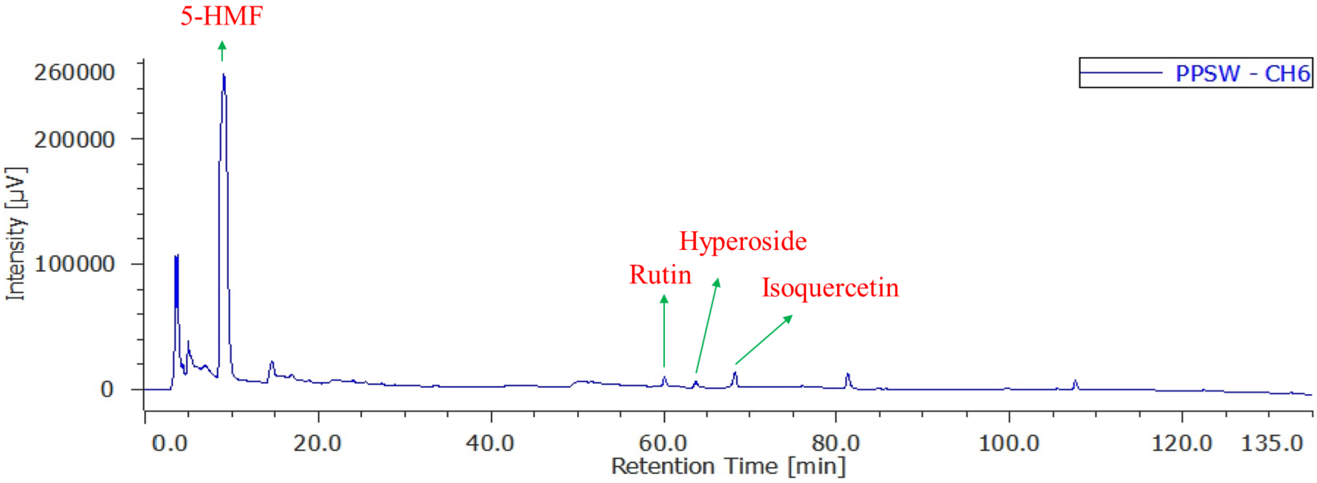

Figure S13. Reversed-phase HPLC chromatogram of water extract in PPS.

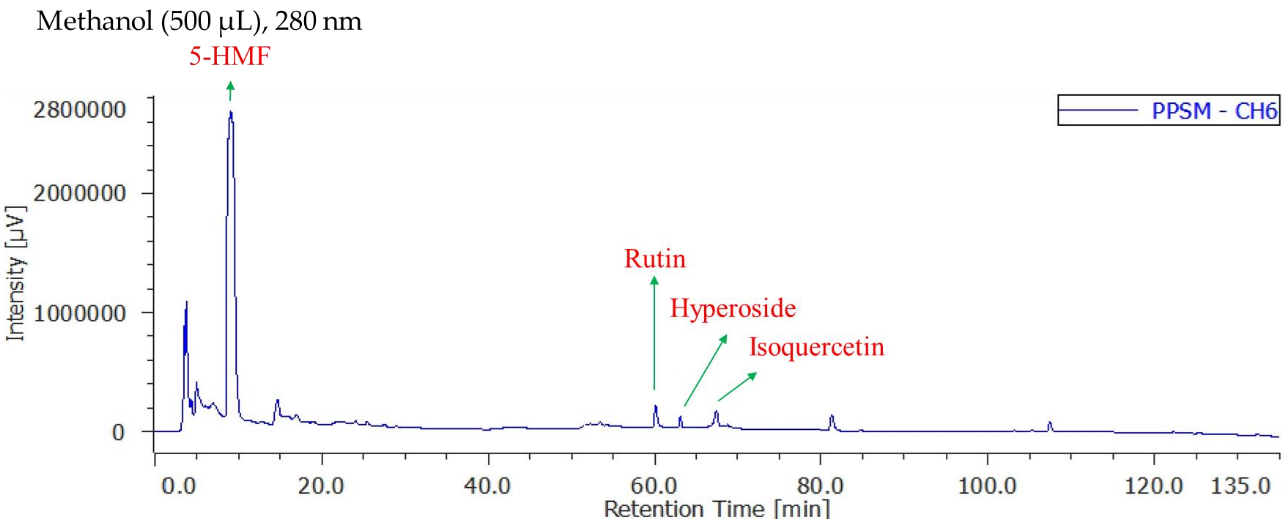

Figure S14. Reversed-phase HPLC chromatogram of methanol extract in PPS.

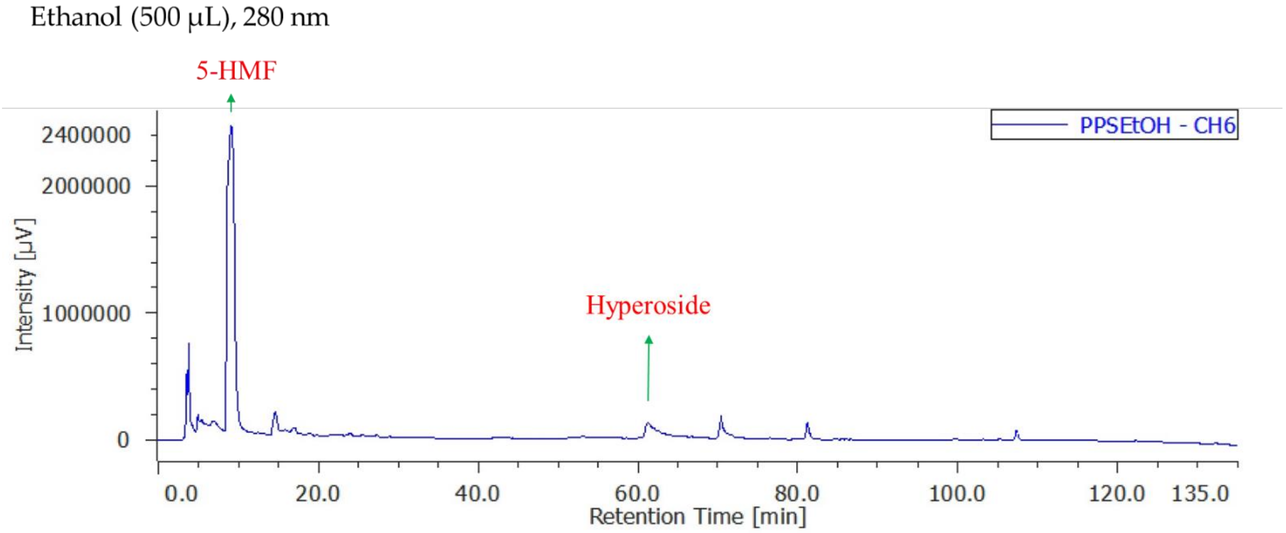

Figure S15. Reversed-phase HPLC chromatogram of ethanol extract in PPS.

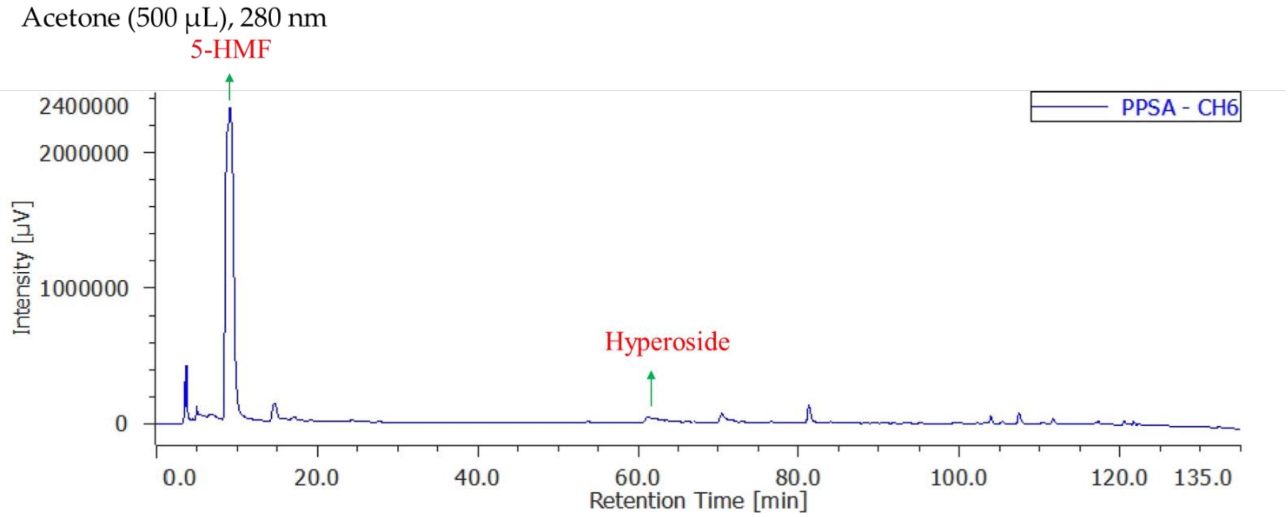

Figure S16. Reversed-phase HPLC chromatogram of acetone extract in PPS.

Ethyl acetate (500 µL), 280 nm

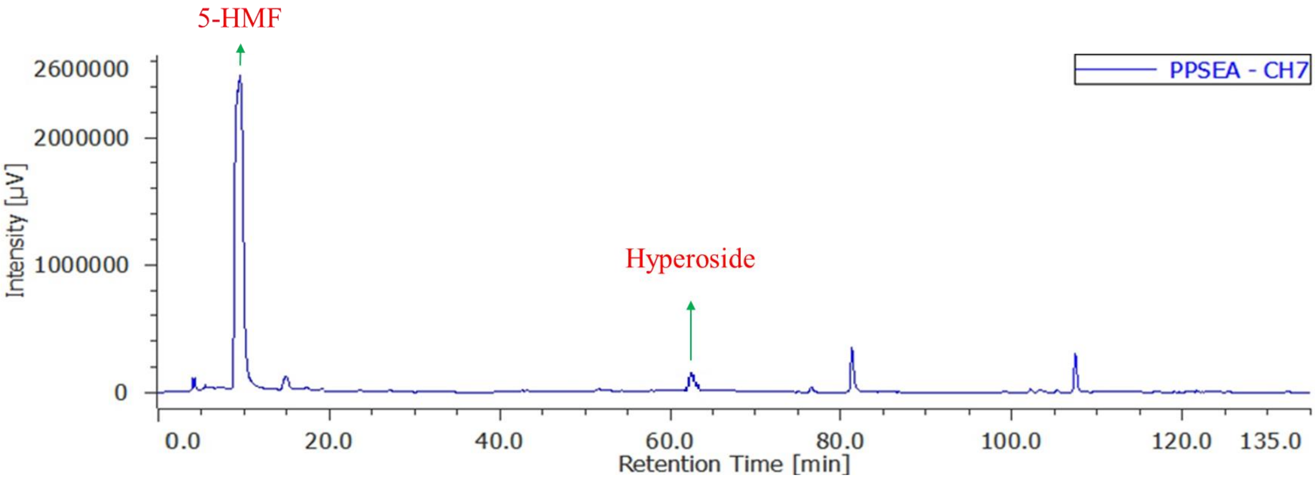

Figure S17. Reversed-phase HPLC chromatogram of ethyl acetate extract in PPS.

Dichloromethane (500 µL), 280 nm

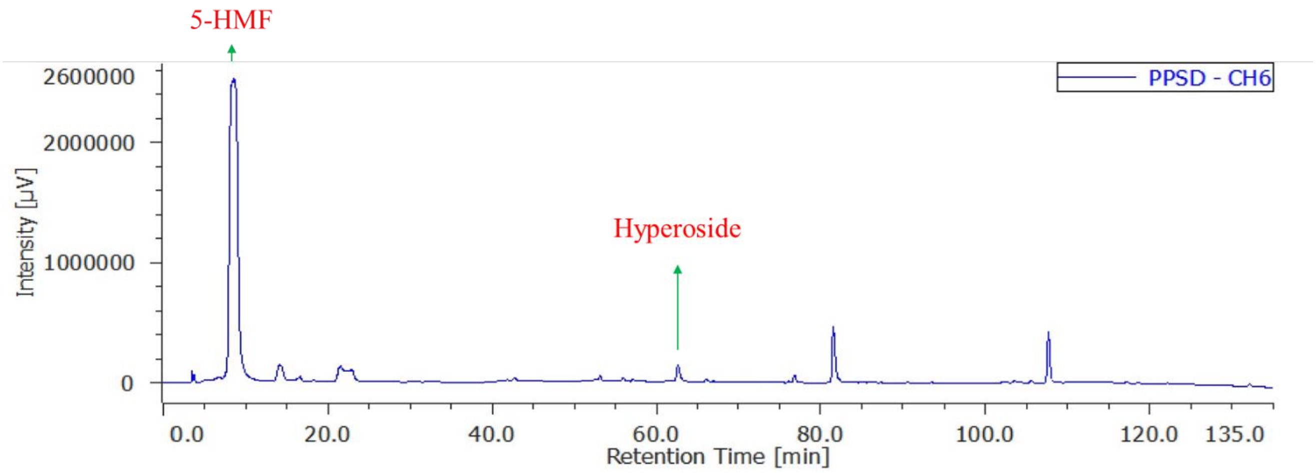

Figure S18. Reversed-phase HPLC chromatogram of dichoromethane extract in PPS.

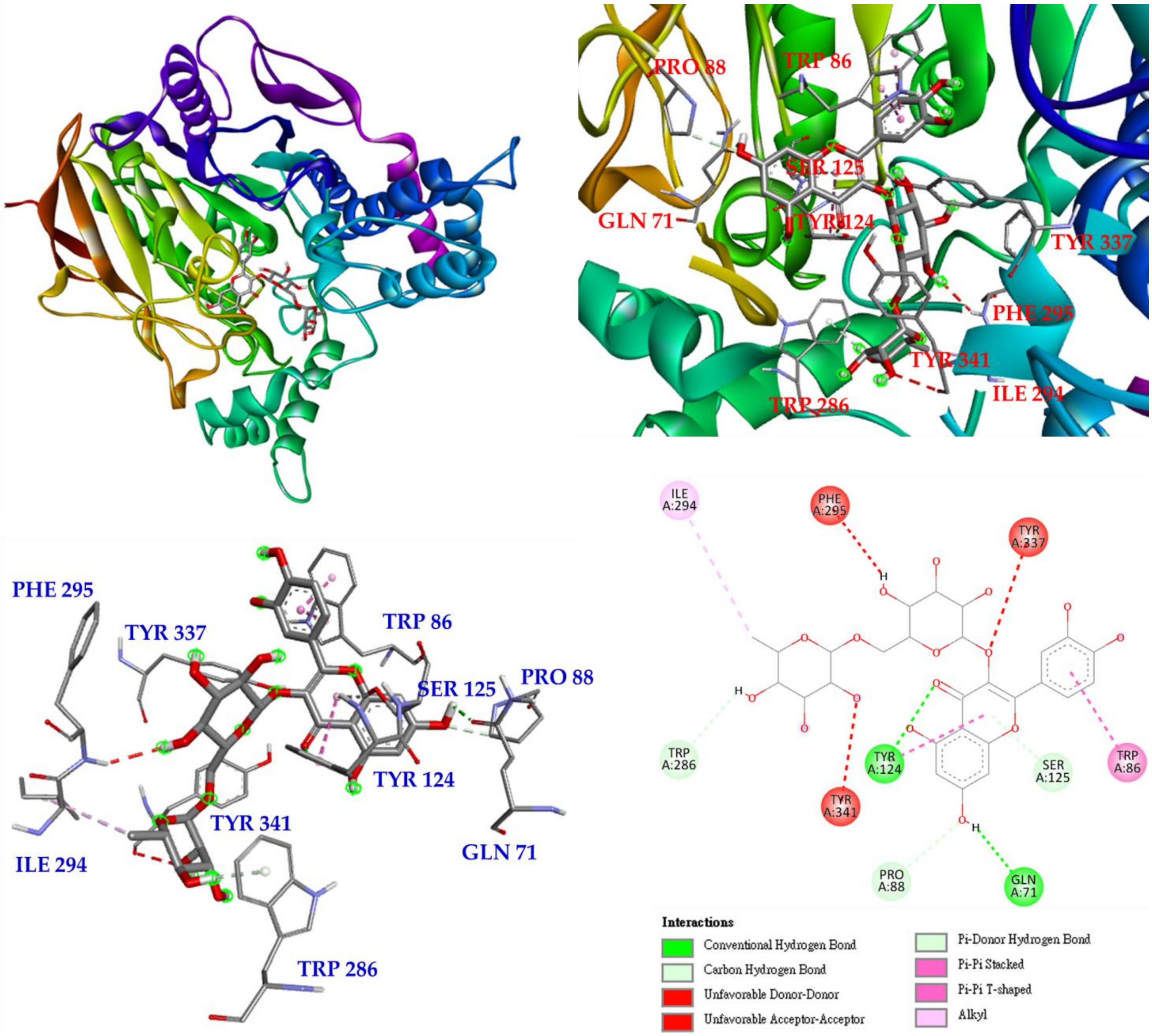

Figure S19. Interactions of rutin (**5**) with active sites of *E. electricus* AChE.
